# Anti-Calcification Effects of Immortalized Human Adipose Tissue-Derived Mesenchymal Stem/Stromal Cell (HATMSC2) Secretome in an In Vitro Model of Vascular Calcification

**DOI:** 10.3390/cells15211925

**Published:** 2026-10-23

**Authors:** Paulina Piotrowska, Honorata Kraskiewicz, Jolanta Lis-Kuberka, Aleksandra Klimczak

**Affiliations:** Laboratory of Biology of Stem and Neoplastic Cells, Hirszfeld Institute of Immunology and Experimental Therapy, Polish Academy of Sciences, R. Weigla 12, 53-114 Wroclaw, Poland; paulina.piotrowska@hirszfeld.pl (P.P.); honorata.kraskiewicz@hirszfeld.pl (H.K.); jolanta.lis-kuberka@hirszfeld.pl (J.L.-K.)

**Keywords:** vascular calcification, vascular smooth muscle cells (VSMCs), mesenchymal stem/stromal cell secretome, anti-calcification, calcification inhibitors

## Abstract

Vascular calcification (VC) is a very common complication associated with aging, chronic kidney disease, diabetes, and atherosclerosis, predicting cardiovascular morbidity and mortality, but remains largely resistant to current therapies. This study investigated the anti-calcification properties of a secretome derived from a human adipose tissue-derived mesenchymal stem/stromal cell line (HATMSC2) using an in vitro model of vascular smooth muscle cell (VSMC) calcification. VSMCs were cultured in both inorganic and organic calcification media with or without HATMSC2 secretome supplementation (50 μg/mL). Calcium deposition was assessed with Alizarin Red S staining, while osteogenic markers (*RUNX2*, *OPN*, *OSX*, *COL-1*, *OCN*) and contractile marker α-SMA were evaluated with Western blot and RT-PCR. The HATMSC2 secretome significantly reduced calcium deposits and suppressed the upregulation of osteogenic markers in the organic calcification model, preventing osteogenic transdifferentiation. Furthermore, the secretome significantly decreased the adhesion of the THP-1 monocyte to VSMCs under calcification-promoting conditions, confirming its immunomodulatory effects. The detection of BMP antagonists, GREM1 and CHRDL1, MGP (anti-calcification factor), and the BMP signaling agonist BMP-7 in the HATMSC2-derived secretome indicates factors that may potentially contribute to its anti-calcification activity. These findings are consistent with the modulation of osteogenic signaling pathways; however, the precise molecular mechanisms and the individual contribution of the identified proteins require further functional validation.

## 1. Introduction

Vascular calcification (VC) is a major public health burden in aging populations; however, it can also develop relatively early in individuals with chronic kidney disease (CKD), diabetes mellitus, and atherosclerosis. VC predominantly affects individuals over 65 years of age, driven by age-related oxidative stress and chronic low-grade inflammation [1,2]. VC is strongly associated with arterial stiffness, which translates into impaired cardio-vascular hemodynamics [3]. As a complex pathophysiological condition, VC contributes to hypertension, reduced organ perfusion, and severe cardiovascular complications. Because the occurrence of VC serves as an independent predictor of cardiovascular morbidity and mortality, identifying effective therapeutic strategies remains a priority [4,5].

VC is an active, tightly regulated process that mimics osteogenesis and chondrogenesis at both functional and molecular levels, encompassing passive mineral deposition and active cellular transformation. Passive deposition involves the pathological accumulation of calcium salts, primarily hydroxyapatite, within blood vessel walls, causing stiffening and loss of elasticity [6,7]. This is caused by unregulated calcium-phosphate imbalances (e.g., elevated phosphate levels) and promotes the formation of calciprotein particles I and II (CPP I/II) without direct cellular control [8]. The active processes of VC include the phenotypic switching of vascular smooth muscle cells (VSMCs) toward an osteo/chondrogenic phenotype, marked by the downregulation of contractile markers, such as smooth muscle α-actin (α-SMA), smooth muscle myosin heavy chain (SM-MHC), smooth muscle protein 22α (SM22α), and calponin [9,10]. Simultaneously, pathological calcification drives the upregulation of the runt-related transcription factor 2 (*RUNX2*), a master osteogenic transcription factor that promotes matrix mineralization and the expression of bone-related proteins [11,12,13]. As previously reported, *RUNX2* directly activates the secreted phosphoprotein 1 (*SPP1*) gene to upregulate osteopontin (*OPN*), a multifunctional phosphoprotein that modulates VSMCs phenotypic switching, macrophage infiltration, and osteoclast formation in calcified lesions. However, the role of OPN may be context-dependent (pro- vs. anti-calcification), and these divergent effects are strictly determined by microenvironmental factors [14,15]. This is consistent with in vivo models in which high phosphate feeding induces arterial medial calcification in uremic rats, an effect that can be attenuated by the phosphate binder lanthanum carbonate [16]. Specifically, in VSMCs undergoing phenotypic switching toward an osteogenic state during pathological calcification, OPN modulates this transition by altering extracellular matrix interactions and signaling (e.g., via integrin receptors), while also recruiting macrophages to inflamed lesions and influencing osteoclast-like activity that can either amplify or resolve mineral deposition [17,18]. VC substantially reduces quality of life and imposes considerable costs on healthcare systems. However, current pharmacological interventions remain largely supportive, and no gold-standard therapy exists that effectively prevents or reverses VC, underscoring an urgent need for innovative disease-modifying strategies that directly target the calcification process, rather than its downstream consequences [19].

In recent years, mesenchymal stem/stromal cells (MSCs), and particularly their secretome, have emerged as a promising strategy for cardiovascular regeneration and tissue repair [20,21,22]. However, most of the available experimental evidence on the MSC-derived secretome in VC originates from studies using bone marrow-derived MSCs (BM-MSCs), underlining the need to explore alternative MSC sources, such as human adipose tissue-derived lines [23,24,25,26,27]. Human adipose tissue-derived MSCs (HATMSCs) are particularly attractive due to their high availability, minimally invasive harvesting procedures, and potent immunomodulatory, proangiogenic, and regenerative properties [28,29]. The secretome of HATMSCs contains a rich repertoire of bioactive molecules, including growth factors (such as vascular endothelial growth factor (VEGF), hepatocyte growth factor (HGF), insulin-like growth factor 1 (IGF-1), and transforming growth factor-beta (TGF-β)), cytokines, chemokines, molecules involved in remodeling of the extracellular matrix (matrix metalloproteinases (MMP)), and extracellular vesicles loaded with regulatory proteins, lipids, and non-coding RNAs, which collectively modulate inflammation, angiogenesis, apoptosis, and osteogenic signaling in the vascular wall [28,30,31,32].

To the best of our knowledge, the anti-calcification potential of the secretome obtained from immortalized HATMSC lines has not been investigated to date. Therefore, in this study, we have focused on evaluating the anti-calcification potential of the secretome obtained from our immortalized human adipose tissue-derived mesenchymal/stromal stem cells (HATMSC2). We have previously demonstrated that the HATMSC2 secretome exerts a pro-regenerative effect on skin-derived cells in an in vitro wound model [30,33]. Given the complexity of the molecular machinery underlying VC, encompassing passive mineral deposition, active VSMC osteogenic switching, and context-dependent regulatory proteins such as OPN and RUNX2, there is a compelling rationale for identifying therapeutic strategies capable of modulating multiple nodes of this cascade simultaneously. In this context, the paracrine activity of MSCs has emerged as a particularly promising avenue, offering pleiotropic anti-calcification effects through the secretion of bioactive molecules that target key osteogenic pathways. Growing evidence supports a protective role of MSCs in VC, acting predominantly through paracrine mechanisms, including the secretion of soluble factors [25,34], extracellular vesicles, and exosomal miRNAs [35,36], rather than through direct osteogenic differentiation.

We hypothesize that the HATMSC2-derived secretome inhibits VC in VSMCs by suppressing osteogenic differentiation and mineralization through the modulation of key osteogenic signaling pathways. This study aimed to evaluate the anti-calcification potential of the HATMSC2-derived secretome in an in vitro model of vascular calcification and to investigate the molecular mechanism of this process by characterizing changes in the VSMC phenotype through an assessment of osteogenic markers and inflammatory cell adhesion. In addition, we attempted to identify soluble protein components that may contribute to its anti-calcification effects.

## 2. Materials and Methods

### 2.1. Cell Culture

Each cell line used in the study was cultured in a dedicated cell culture medium supplemented with 1 mM L-glutamine, 100 IU/mL penicillin, and 100 μg/mL streptomycin. Human aortic vascular smooth muscle cells (VSMC; ATCC, Manassas, VA, USA) were cultured in Dulbecco’s Modified Eagle Medium (DMEM, IIET PAS) supplemented with 10% Fetal Bovine Serum (FBS). The human leukemia monocytic cell line Tohoku Hospital Pediatrics-1 (THP-1; ATCC, Manassas, VA, USA) was cultured in the Roswell Park Memorial Institute 1640 (RPMI-1640, IIET PAS) medium enriched with 10% FBS and with 0.05 mM of 2-Mercaptoethanol (Thermo Fisher Scientific, Gibco, Grand Island, NY, USA).

### 2.2. Production of Secretome from HATMSC2

The secretome was produced using the HATMSC2 cell line, an immortalized human adipose tissue-derived mesenchymal stem cell line established in our laboratory from primary atMSCs obtained from a healthy donor. The immortalization procedure was performed through a stable genetic modification of primary atMSCs with pSV3-neo and hTERT plasmids using the ViaFect™ Transfection Reagent (Promega, Mannheim, Germany). Briefly, primary atMSCs were transfected with the respective plasmids and subsequently selected using Geneticin (for cells transfected with pSV3-neo) and Puromycin (for cells transfected with hTERT). The selection was performed for approximately three weeks, with a gradual increase in antibiotic concentrations to obtain stable cell populations. Following successful selection, the resulting immortalized, non-tumorigenic atMSCs were established as the HATMSC2 cell line. The detailed procedure for the generation and characterization of the HATMSC2 cell line was described in our previous paper [30].

The HATMSC2 secretome was produced according to our previously described protocol [30]. In brief, HATMSC2 cells were seeded in T500 flasks (Thermo Scientific, Roskilde, Denmark) at a density of 2 × 10^4^ cells/cm^2^ in DMEM supplemented with 10% FBS. After 24 h of incubation at 37 °C and 5% CO_2_, the culture medium was removed, and the culture surface was rinsed with DMEM for 10 min at room temperature (RT) to wash any remaining serum from the cells. The cells were then cultured for a further 24 h under ischemic conditions (1% O_2_, 5% CO_2_, serum-free medium), after which the conditioned medium was collected and centrifuged twice for 10 min at 1600 rcf to remove cellular debris. The resulting native secretome from HATMSC2 was concentrated 20-fold (*v*/*v*) using Amicon^®^ Ultra 15 mL Centrifugal 3 kDa filters (Merck Millipore, Carrigtwohill, Ireland). The total protein concentration in both the native and concentrated secretome samples was determined with the Bradford method using a Bio-Rad kit (Bio-Rad, Munich, Germany). Samples were stored at −20 °C prior to use. For all experiments, the HATMSC2 secretome was prepared from a minimum of two independent secretome batches.

### 2.3. In Vitro Calcification of Vascular Smooth Muscle Cells

To mimic the microenvironment typical of vascular calcification, VSMCs were exposed to two in vitro calcification models, utilizing inorganic and organic phosphate donors, respectively.

#### 2.3.1. Inorganic Calcification Model

The VSMCs were seeded into 24-well plates at a density of 6 × 10^3^ cells per well, using minimum essential medium (MEM) supplemented with 10% FBS. Following 24 h of culture under standard conditions (37 °C, 21% O_2_, 5% CO_2_), the medium was replaced with MEM containing 0.1% FBS and 2 mM phosphate, as previously described [37]. In brief, two sodium phosphate buffer solutions were prepared: 1 M NaH_2_PO_4_ (pH ~4.6) and Na_2_HPO_4_ (pH ~9.0). Next, a calcification buffer was prepared by adding 387 µL of 1 M NaH_2_PO_4_ and 113 µL of 1 M Na_2_HPO_4_ to 4500 µL of distilled water. The resulting solution was sterilized by filtration using a 0.22 µm syringe filter. The calcification medium was prepared by adding 1.3 mL of a calcification buffer to 63.7 mL of MEM containing 0.1% FBS, 1 mM L-glutamine, 100 IU/mL penicillin, and 100 μg/mL streptomycin. The cells were cultured for up to 14 days with the medium replaced every two days. Cells maintained in MEM supplemented with 0.1% FBS were used as the non-calcifying control (C−). To assess the effect of the HATMSC2 secretome, concentrated secretome preparations, quantified as total protein, were added to the calcification medium at final concentration of 20 µg/mL (C+S20+) and 50 µg/mL (C+S50+). At selected time points (days 3, 9, 11, and 14), the medium was collected, and the cells were washed with pre-warmed phosphate-buffered saline (PBS) and fixed with 4% paraformaldehyde (PFA) for 20 min. The cells were stored in PBS at 4 °C for further analysis.

#### 2.3.2. Organic Calcification Model

The organic calcification model was established using DMEM supplemented with 10% FBS and calcification agents, such as 10 mM β-glycerophosphate (β-GP), 50 µM ascorbic acid, and 0.1. µM dexamethasone. VSMCs were seeded at a density of 3 × 10^3^ cells per well into 24-well plates in the basal culture medium, and after 2 h of incubation under standard conditions (37 °C, 21% O_2_, 5% CO_2_), the medium was replaced with the calcification medium. Due to the nature of the organic model, which requires a long time to induce calcification, the culture was maintained for up to 21 days. In this setup, the non-calcifying control (C−) consisted of VSMCs cultured in DMEM supplemented with 10% FBS, and the cells were passaged every 2–3 days due to their rapid proliferation associated with the high serum content. The positive calcification control (C+) consisted of cells cultured in an organic calcification medium. As in the inorganic model, experimental groups were established in which the calcification medium was supplemented with HATMSC2-derived secretome preparations, and normalized to total protein at a final concentration of 20 µg/mL (C+S20+) and 50 µg/mL (C+S50+). Cells in the calcifying medium groups (C+ and C+S50+) were not passaged throughout the experiment to avoid disrupting the deposited calcium mineral. At selected time points (days 3, 6, 9, 14, and 21), the medium was collected, and the cells were washed with pre-warmed PBS and fixed with 4% PFA for 20 min. The cells were then stored in PBS at 4 °C for further analysis.

### 2.4. Comparison of Cell Viability Between Calcification Models Assessed with SRB Assay

To evaluate VSMC viability in inorganic (MEM with 0.1% FBS, containing 2 mM phosphate) and organic (DMEM supplemented with 10% FBS and enriched with 10 mM β-GP) calcifying media, the total cellular protein content was measured using the SRB assay. Cells were seeded into 96-well plates at a density of 1 × 10^3^ cells per well in the basal culture medium for each calcification model (MEM supplemented with 0.1% of FBS in the inorganic calcification model, and DMEM supplemented with 10% of FBS in the organic calcification model). After 2 h, the medium was changed for the specific model for both the C+ groups and those supplemented with the HATMSC2-derived secretome (C+S50+).

At selected time points, the cells were washed with PBS and then fixed in a 50% trichloroacetic acid solution for 1 h at 4 °C. After fixation, the samples were washed with distilled water and left to dry at RT. The plates were then incubated with a 0.4% SRB solution for 30 min in the dark. Excess dye was removed by washing with 1% acetic acid, and the plates were dried again. The protein-bound dye was dissolved in a 10 mM TRIS solution, and the plates were shaken for 30 min. Absorbance was measured using a Wallac Victor2 microplate reader (PerkinElmer, Waltham, MA, USA) with a wavelength of 570 nm.

### 2.5. Alizarin Red S Staining and Quantification

The presence of calcium phosphate deposits in VSMCs subjected to in vitro calcification was assessed using Alizarin Red S staining. At selected time points, the cells were washed with PBS, then fixed in 10% formalin for 20 min at RT and washed again with PBS. Subsequently, 200 µL of a 2% solution of Alizarin Red S (Sigma-Aldrich, St. Louis, MO, USA) was added to each well and incubated for 10 min at RT. The wells were rinsed with PBS, and images were taken using a Zeiss microscope (Carl Zeiss, Jena, Germany). Cetylpiridinium chloride (CPC) was used for the quantitative analysis of calcium phosphate deposits. After removing the PBS, the cells were incubated with 200 µL of a 10% CPC solution (Sigma-Aldrich, St. Louis, MO, USA) at 37 °C for 2 h. Subsequently, 100 µL of the resulting solution was transferred to a 96-well plate, and absorbance was measured at 405 nm using a Wallac Victor2 microplate reader (PerkinElmer, Waltham, MA, USA).

### 2.6. Alkaline Phosphatase Activity

Alkaline phosphatase (ALP) activity was measured using an ALP assay kit (Sigma-Aldrich, St. Louis, MO, USA). VSMCs were subjected to two in vitro calcification models, in accordance with the conditions set out in Table 1. At selected time points, 50 µL of culture medium was collected and stored at −20 °C before further analysis. All samples were analyzed simultaneously, according to the manufacturer’s instructions. Optical density measurements were performed at a wavelength of 405 nm using a CLARIOstar^®^ microplate reader (BMG LABTECH, Ortenberg, Germany).

### 2.7. THP-1 Adhesion Assay

The pro-inflammatory activity of VSMCs induced by the calcification process was assessed with the THP-1 human monocytic cell line adhesion assay, based on the protocol designed by Pang et al. 2021 [38], with minor modifications. Briefly, 1.5 μL of DiO dye (live-cell staining; cat. no. V22889, Life Technologies, Carlsbad, CA, USA) was added to 1.5 mL of a THP-1 cell suspension at a concentration of 1.5 million cells per mL of PBS, and the mixture was incubated for 7 min at 37 °C. The THP-1 cells were then resuspended in DMEM supplemented with 10% FBS and centrifuged at 1200 rcf, after which the cells were resuspended in fresh DMEM. A total of 1 × 10^5^ THP-1 cells were added to VSMCs cultured in a 48-well plate in the basal cell medium (C−), organic calcification medium alone (C+), and calcification medium enriched with HATMSC2 secretome (C+S50+). The co-culture was incubated for 1 h at 37 °C, then the medium was removed, and the wells were washed three times with pre-warmed PBS to remove unbound THP-1 cells. Images of five random fields of each well were taken using an inverted Axio Observer fluorescence microscope (Zeiss, Jena, Germany) with a 5× objective, and the number of the bound THP-1 cells was counted using the microscope’s dedicated Zeiss Zen Blue software, v3.11 (Carl Zeiss, Jena, Germany). The number of adherent DiO-stained THP-1 cells was determined manually by marking each DiO-positive cell in the captured images. Prior to analysis, the images were coded and randomized, and the analysis itself was performed by a researcher blinded to the experimental group assignments.

### 2.8. Real-Time PCR for Osteogenic Gene Expression

To assess the expression of genes associated with osteogenesis in VSMCs, both early markers, including osterix (*OSX*), bone morphogenetic protein 2 (*BMP-2*) and collagen type I (*COL-1*), as well as late markers, osteocalcin (*OCN*) and *OPN*, were analyzed (Table 2). The VSMCs were cultured in T25 flasks in three different conditions: the basal culture medium (DMEM supplemented with FBS for a final concentration of 10%) and both calcification media alone (basal medium enriched with 10 mM β-GP, 50 µM ascorbic acid, and 0.1 µM dexamethasone) and supplemented with HATMSC2 secretome for a final concentration of 50 µg/mL. On days 3, 6, 9, 14, and 21, the cells were treated with trypsin and rinsed twice with the PBS buffer. Total RNA was then isolated and purified using the NucleoSpin RNA kit (MACHEREY-NAGEL GmbH & Co. KG, Düren, Germany), in accordance with the manufacturer’s instructions. Reverse transcription of 1 µg of total RNA from each sample was carried out using the First Strand cDNA Synthesis Kit (Thermo Fisher Scientific Baltics UAB, Vilnius, Lithuania). The Power SYBR Green PCR Master Mix (Life Technologies LTD, Warrington, UK) was used to perform real-time PCR, and the increase in fluorescence signal was monitored using the ViiA 7 Real-Time PCR System (Applied Biosystems, Foster City, CA, USA). The reaction protocol involved an initial denaturation at 95 °C for 10 min, followed by 40 cycles consisting of denaturation at 95 °C for 15 s, annealing at 55 °C for 1 min, and extension at 72 °C for 40 s. The reference gene encoding hypoxanthine-guanine phosphoribosyltransferase (HPRT) was used to normalize qPCR product quantities (∆CT), while relative mRNA expression levels were calculated using the 2^−∆∆CT^ method. The results are presented as the mean (SD or SEM) from three independent experiments, each of which was performed in triplicate.

### 2.9. Western Blot Analysis

Selected osteogenic (RUNX2, OPN) and contractile (α-SMA) markers were evaluated at the protein level with β-actin used as an internal loading control. Following SDS-PAGE, the protein samples, along with positive controls (HepG2 and Saos-2 cells lysate, please see Supplementary Figure S1), were transferred to PVDF membranes (Merck KGaA, Darmstadt, Germany; Sigma-Aldrich, St. Louis, MO, USA). The membranes were blocked with a blocking buffer (Bio-Rad Laboratories, Hercules, CA, USA) for 10 min at RT. Incubation with primary antibodies was carried out overnight at 4 °C with antibodies diluted 1:1000, including affinity-purified goat anti-OPN IgG (cat. no. AF1433, R&D Systems, Minneapolis, MN, USA), rabbit anti-RUNX2 IgG (cat. no. 12556S, Lot no.: 5; Cell Signaling Technology, Danvers, MA, USA), mouse anti-human α-smooth muscle cell actin (α-SMA, cat. no. 14-9760-82, Lot no.: 3031637; Thermo Fisher Scientific, Waltham, MA, USA) and rabbit anti-β-actin (cat. no. 4970S, Lot no.: 19; Cell Signaling Technology, Danvers, MA, USA) as a loading control. Secondary antibodies were applied as follows: goat anti-immunoglobulin (cat. no. E0466, Lot no.: 038(201); Dako, Glostrup, Denmark; 1:5000, 1 h at RT) for OPN, followed by the streptavidin-HRP conjugate (cat. no. P0397, Lot no.: 20004716; Dako, Glostrup, Denmark; 1:12,500) for signal amplification, goat anti-rabbit IgG-HRP (cat. no. AC2114, Lot no.: 241025-87; Azure Biosystems, Dublin, CA, USA; 1:40,000, 1 h at RT) for RUNX2 and β-actin, and goat anti-mouse IgG-HRP (cat. no. AC2115, Lot no.: 240216-41; Azure Biosystems, Dublin, CA, USA; 1:10,000, 1 h at RT). The specificity of all secondary antibodies was confirmed during method optimization by demonstrating the lack of a detectable signal in the absence of the primary antibody. Chemiluminescent signals were detected using the SuperSignal West reagent (Thermo Fisher Scientific, Waltham, MA, USA). Western blot results are expressed as the mean ± SD values from three independent experiments for OPN and RUNX2 and two for α-SMA. Band intensities, reflecting antibody reactivity to target proteins, were quantified with densitometry using the myImageAnalysis software, v2.0 (Thermo Fisher Scientific, Waltham, MA, USA). β-actin was used as a loading control for the normalization of RUNX2, OPN, and α-SMA protein relative abundance. Densitometric analysis was performed by calculating the ratio of the target-protein band intensity and the intensity of the corresponding β-actin band. To account for inter-membrane variability, the same C− reference lysate was included on each membrane. For each target protein, the target/β-actin ratio of each experimental sample was then divided by the corresponding ratio of the C− reference sample on the same membrane. The obtained values were expressed relative to the C− reference.

### 2.10. Osteopontin Immunostaining

VSMCs were seeded onto 10-mm diameter coverslips placed in 24-well plates and cultured for up to 21 days under three conditions: standard culture medium (non-calcifying control), calcification-inducing medium (calcification control), and calcifying medium supplemented with the HATMSC2-derived secretome at a final concentration of 50 µg/mL. At selected time points, the cells were fixed in 10% formalin for 20 min, followed by permeabilization using 0.1% Triton X-100 (Avantor Performance Materials Poland, Gliwice, Poland) for 15 min at RT. Non-specific binding sites were blocked through incubation with 1% BSA (Symbios, Gdańsk, Poland) for 40 min at RT. The primary antibody goat anti-human osteopontin (OPN) IgG (cat. no. AF1433, R&D Systems, Minneapolis, MN, USA) was diluted 1:100 in an antibody diluent (Life Technologies Corporation, Eugene, OR, USA), then applied in a volume of 75 µL to each coverslip and incubated for 2 h at RT. After three washes of 5 min each in PBS, the cells were incubated with the secondary antibody: donkey anti-goat IgG AF647 (cat. no. A21447, Lot no. 3034156, Life Technologies Corporation, Eugene, OR, USA) at a dilution of 1:1000 for 1 h at RT, and AF488-conjugated phalloidin (cat. no. 59-6559-05, Lot no. E143276, Invitrogen, Carlsbad, CA, USA) at a 1:500 dilution was used to visualize actin filaments (1 h incubation at RT). The specificity of all secondary antibodies was confirmed during method optimization by demonstrating the absence of a detectable signal in the absence of the primary antibody. Finally, the cover slips were mounted using the SlowFade Gold antifade reagent with DAPI (cat. no. S36939, Lot no. 3268378, Life Technologies Corporation, Eugene, OR, USA). Staining visualization was carried out using an Axio Observer inverted fluorescence microscope with Apotome 3 (Zeiss, Jena, Germany) at 10× and 63× magnifications and analyzed using the Zeiss Zen Blue software, v3.11 (Carl Zeiss, Jena, Germany).

### 2.11. STRING-Based In Silico Identification of Potential HATMSC2 Secretome Proteins Associated with the Attenuation of Vascular Calcification

An in silico protein–protein interaction analysis was performed using the STRING v12.0 database to identify potential functional relationships among the analyzed osteogenic markers and to identify secretome-derived proteins potentially involved in pathways regulating vascular calcification; however, their functional involvement in the anti-calcification effects of the HATMSC2 secretome was not experimentally verified in the present study. The default settings for *Homo sapiens* and a minimum required interaction confidence score of 0.8 were applied. This threshold defined the minimum confidence required for an association between a candidate protein (as a possible down-regulator of vascular calcification) and a target protein (analyzed in the study: OSX, BMP-2, RUNX2, COL-1, α-SMA, OCN, OPN) to be included in the network. The identified associations, together with functional annotations, were used to select candidate proteins for further analysis. Based on the STRING network analysis, three candidates were selected: CHRDL1, GREM1, and MGP. Additionally, BMP-7 and OPG were selected for subsequent quantification in the HATMSC2 secretome by ELISA based on a literature review.

### 2.12. Determination of Potential Secretome Calcification-Related Proteins with ELISA

The concentrations of MGP, CHRDL1, GREM1, BMP-7, and OPG in the HATMSC2 secretome were determined using commercial ELISA kits (Biorbyt, Cambridge, UK) after prior optimization of the assays for this sample type. The assay detection ranges were 0.16–10 ng/mL for OPG, GREM1, and CHRDL1; 15.63–1000 pg/mL for MGP; and 31.25–2000 pg/mL for BMP-7. All samples were analyzed in duplicates. HATMSC2 secretome samples were diluted 5- and 10-fold for GREM1, 250-, and 500-fold for BMP-7, and 500- and 1000-fold for MGP, whereas undiluted samples were used for CHRDL1 and OPG. The intra-assay coefficients of variation were 0.62% for CHRDL1, 7.36% for GREM1, 6.90% for BMP-7, and 3.48% for MGP.

### 2.13. Statistical Analysis

Statistical analyses were performed using GraphPad Prism, v10 (GraphPad Software, California, CA, USA). Data were compared using a two-way analysis of variance (ANOVA) followed by multiple comparison procedures (Dunnet’s test). Values were considered to be significantly different at *p* < 0.05. The level of statistical significance is presented as: * *p* < 0.05; ** *p* < 0.01; *** *p* < 0.001; and **** *p* < 0.0001.

## 3. Results

### 3.1. VSMCs Models of Calcification

To recapitulate the microenvironment of pathological calcification, VSMCs were exposed to two in vitro calcification models, utilizing inorganic and organic phosphate sources, respectively.

#### 3.1.1. Secretome Derived from HATMSC2 Induces a Modest, Non-Significant Reduction in VSMC Calcification in an Inorganic In Vitro Calcification Model

**Figure 1:**
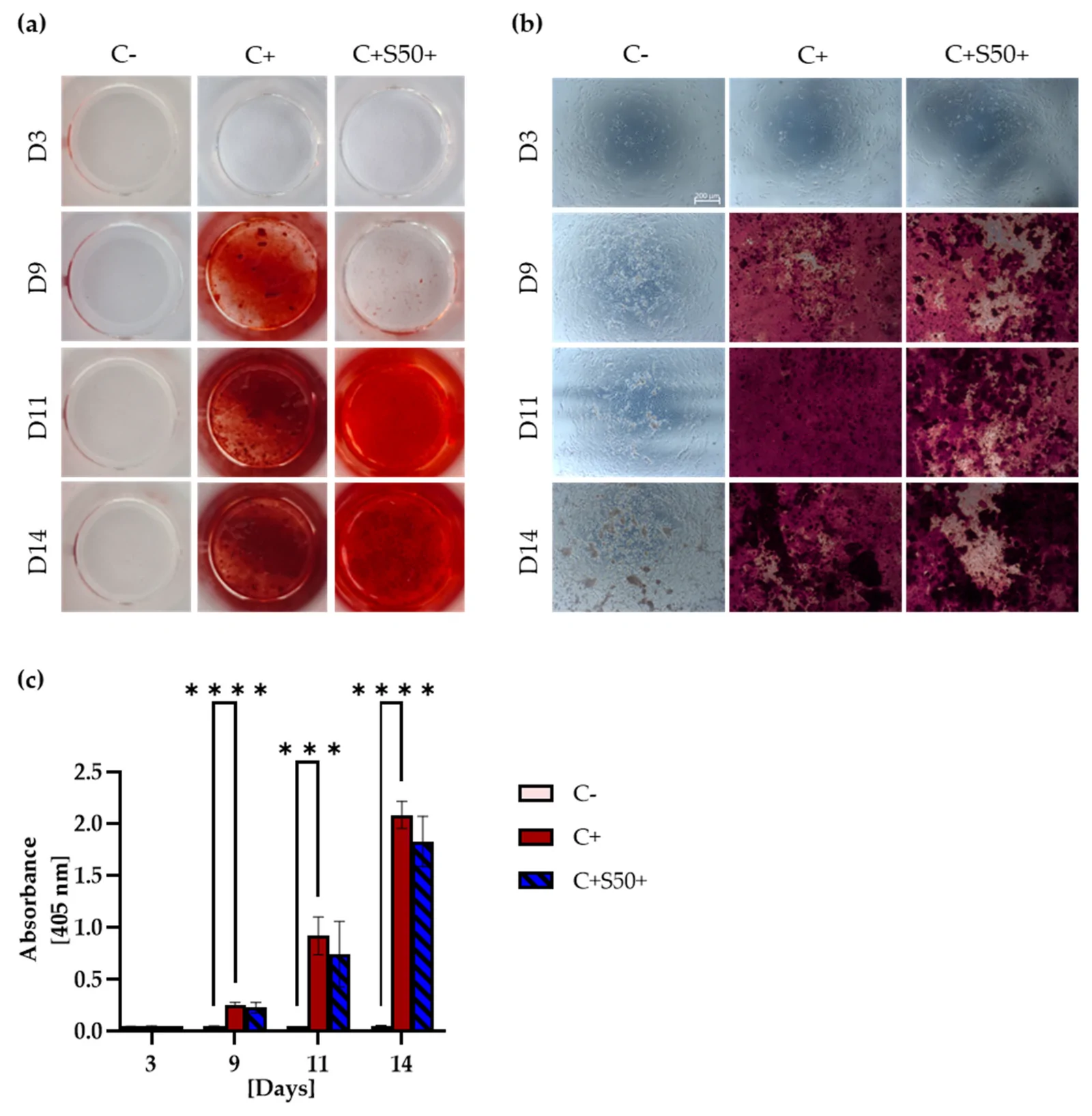
**Calcium deposition in VSMCs cultured in an inorganic calcification model visualized by Alizarin Red S staining.** Representative images of (**a**) macro- and (**b**) microscopic photos (scale bar = 200 µm) of VSMCs cultured in: basal culture medium (C−), calcifying medium alone (C+), and calcifying medium enriched with HATMSC2 secretome at a final protein concentration 50 µg/mL (C+S50+). (**c**) Quantification of Alizarin Red S staining by CPC method. The samples were analyzed in three independent experiments (*n* = 3), each performed in triplicate. Data represent mean ± SD, *** *p* < 0.001, **** *p* < 0.0001. Values were considered significantly different at *p* < 0.05. Statistical significance was compared to C+.

The secretome derived from HATMSC2 induced a modest, non-significant reduction in VSMCS calcification in an inorganic in vitro calcification model Alizarin Red S staining (Figure 1a,b) and its quantification by CPC dissolving (Figure 1c) revealed an increase in the amount of calcium deposits over time in both the positive control group, where the cells were treated with 2 mM inorganic phosphates (C+), and the secretome-treated group (C+S50+). However, the levels of VSMC mineralization were reduced in the secretome-treated group. Throughout the experiment, in the C− group, which consisted of VSMCs cultured in the standard culture medium, no deposits were observed. In contrast, the VSMCs exposed to the inorganic calcification medium (C+) showed strongly stained foci of mineralization. After nine days of the experiment, the absorbance level corresponding to the amount of calcium deposits in the cells subjected to the calcification medium was modestly higher than in the cells cultured in the calcifying medium supplemented with the HATMSC2 secretome (0.25 vs. 0.23, respectively). However, after 11 days, the difference between the two groups increased, with absorbance values reaching 0.92 in the C+ group and 0.74 in the C+S50+ group. Furthermore, the greatest decrease in calcium deposits was observed at the end of the experiment, after 14 days of cell culture, with absorbance values of 2.08 for the C+ group and 1.83 for the C+S50+ group. Despite the clear differences, no statistical significance was demonstrated between these two groups. In addition, when a lower concentration of secretome was used, no anti-calcification effect was observed compared to the C+ control (Supplementary Figure S2a).

#### 3.1.2. Secretome Obtained from HATMSC2 Significantly Reduces VSMC Calcification in an Organic Calcification Model

**Figure 2:**
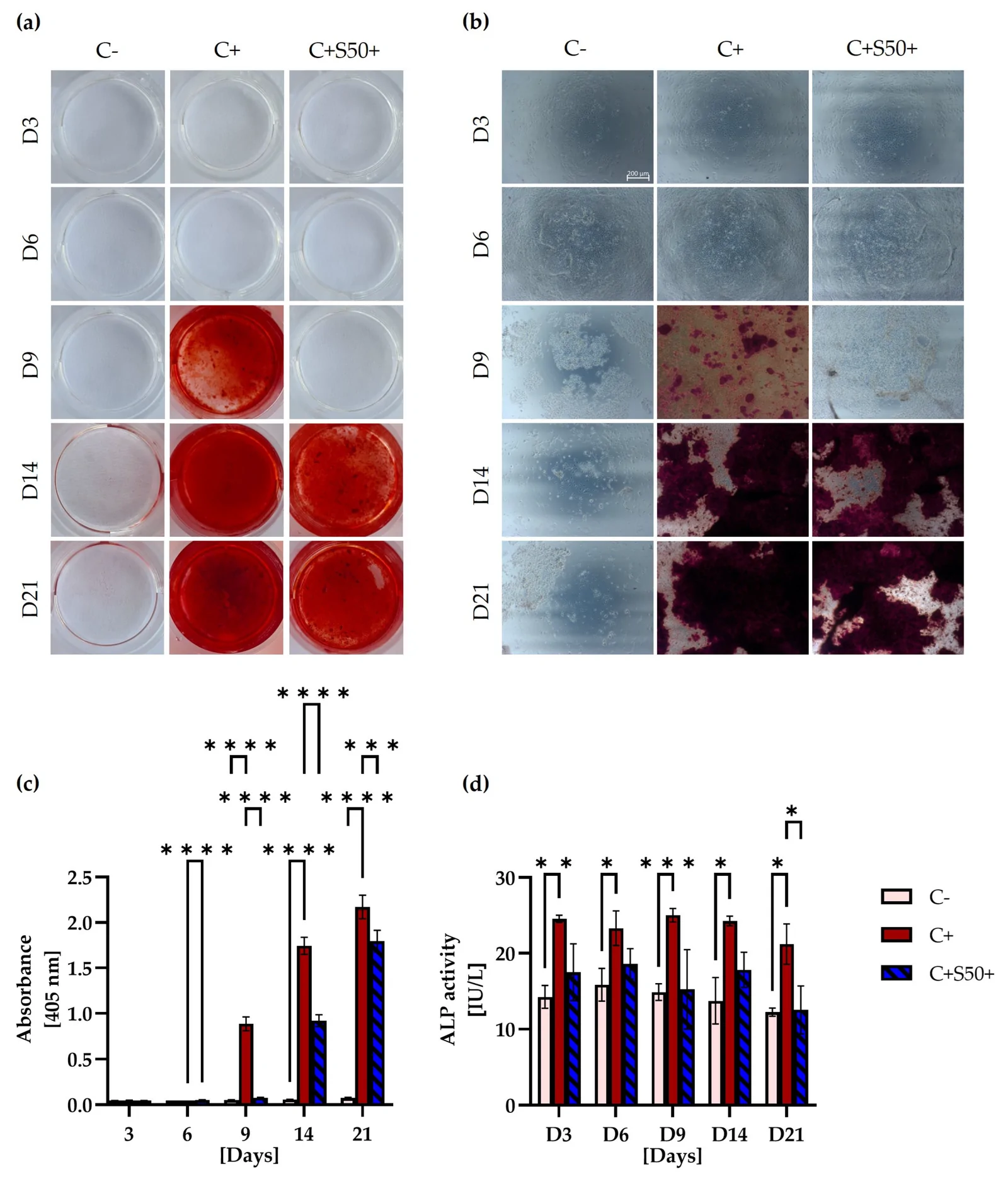
**Calcium deposition and ALP activity in VSMCs cultured in an organic calcification model.** Representative images of (**a**) macro- and (**b**) microscopic photographs of VSMCs following Alizarin Red S staining. Cells cultured in: basal culture medium (C−), calcifying medium (C+), and calcifying medium enriched with HATMSC2 secretome at a final protein concentration 50 µg/mL (C+S50+). (**c**) Quantification of Alizarin Red S staining with the CPC method and (**d**) ALP activity. The samples were analyzed in three independent experiments (*n* = 3), each performed in triplicate. Data represent mean ± SD, * *p* < 0.05, ** *p* < 0.01, *** *p* < 0.001, **** *p* < 0.0001. Values were considered significantly different at *p* < 0.05. Statistical significance was compared to C+.

In the organic calcification model, the first visible changes in calcium deposition occurred on day 9 of the experiment. Alizarin Red S staining revealed an increase in the amount of calcium deposits over time, while no staining was observed in cells cultured in the basic cell medium (C−) (Figure 2a,b). The cells cultured in the organic calcifying medium (C+) showed a statistically significant increase in the amount of calcium deposits as early as day 9 compared to the cells cultured in the basic cell medium (C−) (Abs = 0.89 vs. Abs = 0.05, respectively, *p* < 0.0001) (Figure 2c). Interestingly, at this time point, no calcium deposits were seen in the secretome-treated group (C+S50+), which indicated that supplementation with HATMSC2 secretome inhibited the calcification process in vitro.

After 14 days, an increase in the amount of calcium deposits was observed in the C+S50+ group (Abs = 0.92); however, the absorbance level remained significantly lower than in the C+ group (Abs = 1.74, *p* < 0.0001). On the final day of the experiment, the difference between these two groups decreased. Nevertheless, the group supplemented with the HATMSC2 secretome still exhibited significantly lower absorbance compared to the positive control (Abs = 1.79 vs. Abs = 2.17, respectively, *p* < 0.001). Moreover, when a lower concentration of secretome was used, no anti-calcification effect was observed after 21 days of cell culture compared to the C+ control (Supplementary Figure S2b).

Furthermore, ALP activity showed only minor changes in the C− group (Figure 2d). An increase in enzyme activity was also observed in the cells cultured in the calcifying medium and, interestingly, a decrease in activity was observed in the secretome-enriched group. After 21 days of culture, the level of ALP enzyme activity in the C+S50+ group (12.52 IU/L) was comparable to that in the C− group (12.22 IU/L), and significantly lower than in the C+ group (21.21 IU/L).

### 3.2. VSMCs Exhibit Higher Survival in the Organic Calcification Model than in the Inorganic Calcification Model

**Figure 3:**
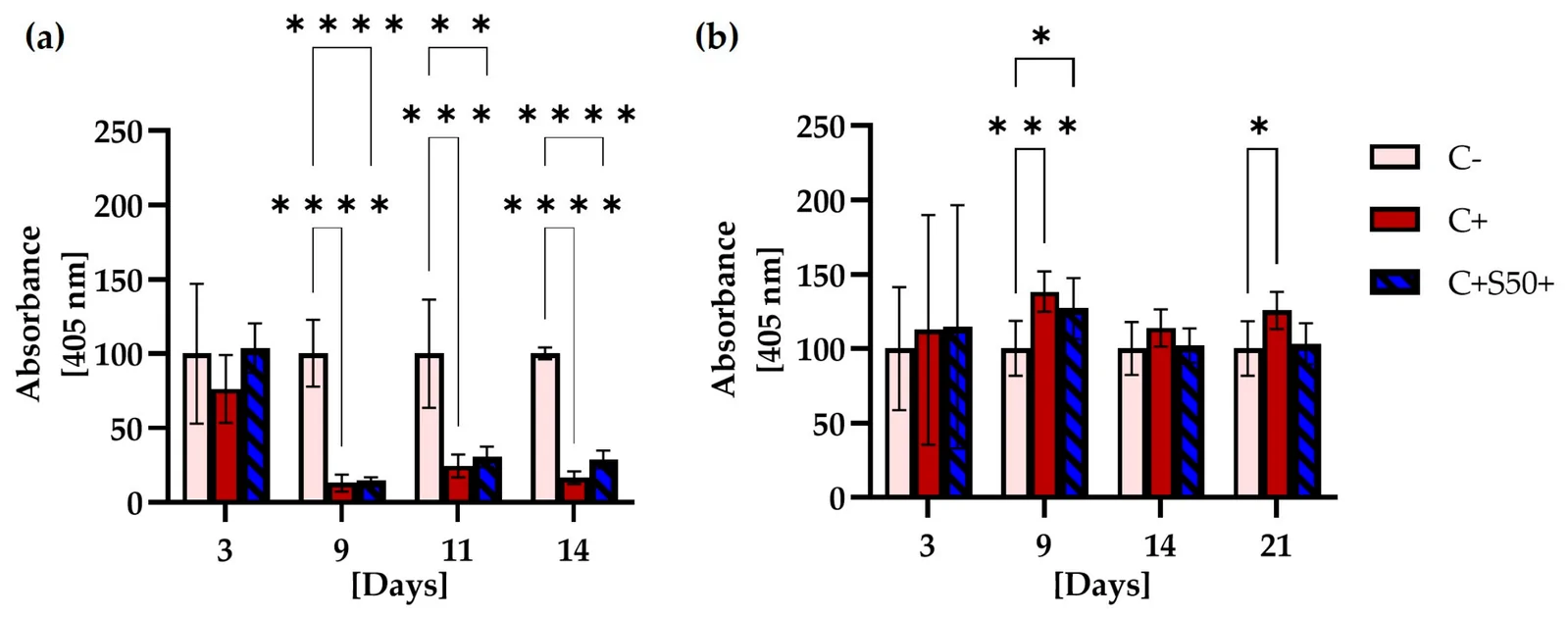
**Viability of VSMCs cultured in an inorganic (a) and organic (b) calcification model.** Cells cultured in: basal culture medium for each model (C−), calcification medium alone (C+), and enriched with HATMSC2 secretome (C+S50+). SRB assay was used to analyze the samples in three independent experiments (*n* = 3), each in triplicate. Data represent mean ± SD, * *p* < 0.05, ** *p* < 0.01, *** *p* < 0.001, **** *p* < 0.0001. Values were considered significantly different at *p* < 0.05. Statistical significance was compared to C−.

Cell viability in both calcification models was assessed using the sulforhodamine B (SRB) assay. In the inorganic calcification model (Figure 3a), VSMC survival was maintained only in the cells cultured in MEM supplemented with 0.1% fetal bovine serum (FBS) without phosphate addition (C−). In contrast, the cells cultured in the medium supplemented with 2 mM inorganic phosphate (C+) and in the medium enriched with the 50 µg/mL HATMSC2 secretome exhibited a statistically significantly lower viability (*p* < 0.0001) compared to the control (C−) at all analyzed time points, except day three, where no significant differences were seen. Especially on day 14, where cell survival ratio fell to 16.5% in the C+ group compared to C−, and 28.8% for C+S50+. The survival rate of the cells from the negative control (C−), i.e., VSMCs cultured in DMEM with 10% FBS, was almost twice as high in the organic model as that of C− in the inorganic model. Interestingly, in contrast to the inorganic model, the results clearly confirmed increased cell survival in the organic calcification model (Figure 3b), where VSMCs cultured for up to 21 days in the calcification medium exhibited statistically significantly (*p* < 0.05) higher viability than the cells cultured in the basic cell medium (C−), (100% and 125.6% respectively). Moreover, the cells cultured in the calcification medium enriched with 50 µg/mL of the HATMSC2 secretome (C+S50+) exhibited a survival rate comparable to that of the cells cultured in basic culture medium, reaching 103.2% after 21 days of cell culture.

### 3.3. Immunomodulatory Activity of HATMSC2 Secretome

**Figure 4:**
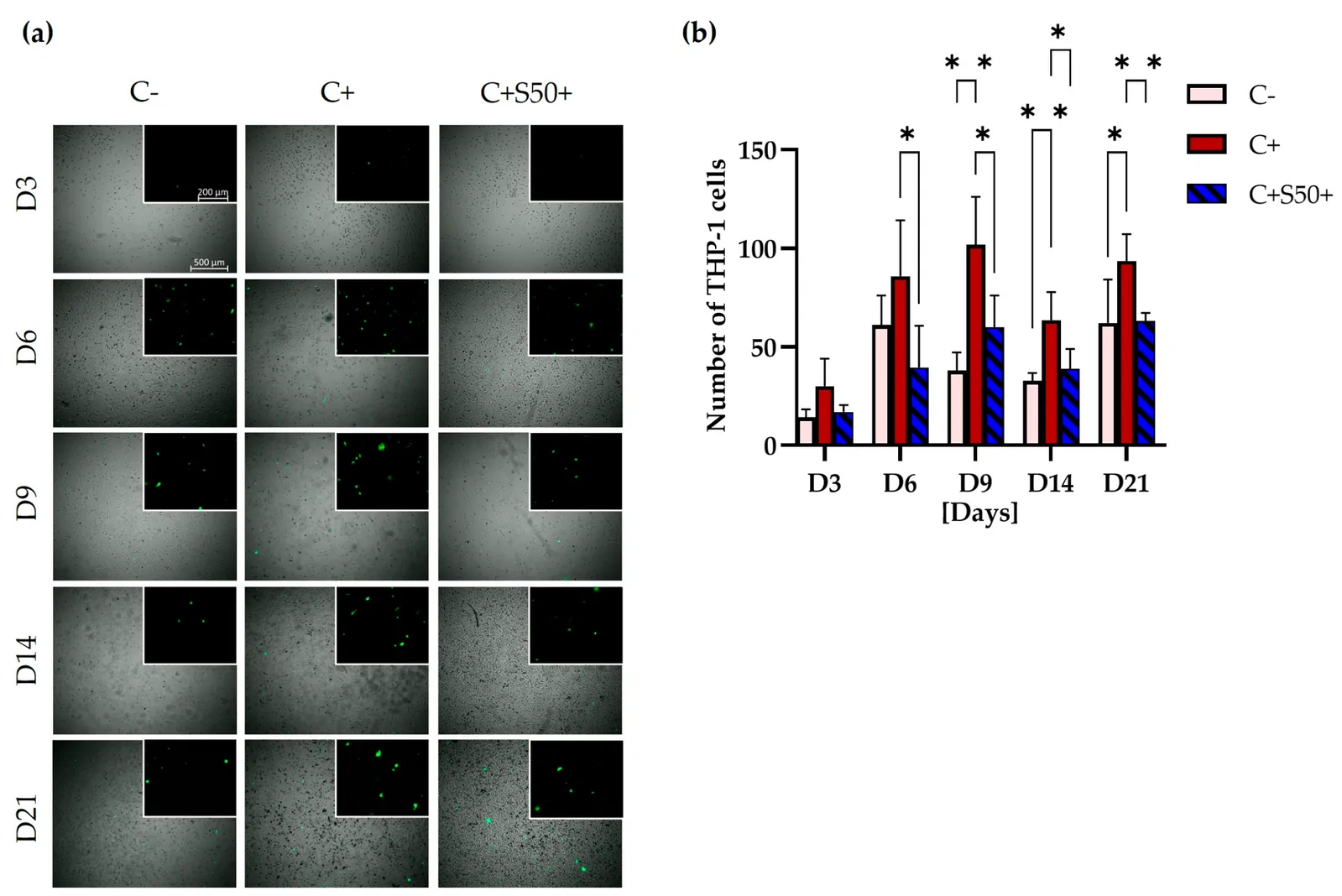
**THP-1 monocyte adhesion to VSMCs in an organic calcification model in vitro.** Cells cultured in: a basal culture medium (C−), organic calcification medium alone (C+), and enriched with HATMSC2 secretome (C+S50+). (**a**) Representative photos of adherent THP-1, (**b**) quantification of THP-1. The samples were obtained from three independent experiments (*n* = 3), each in duplicate. Data represent mean ± SD, *n* = 3, * *p* < 0.05, ** *p* < 0.01. Values were considered significantly different at *p* < 0.05. Statistical significance was compared to C+. Immunofluorescence staining with DiO, brightfield (magnification 5×) and insert (magnification 10×). Scale bars presented in the upper left picture are used for all representative photos. Analyses were performed using the Zeiss Zen Blue software, v3.11.

To evaluate the immunomodulatory potential of the HATMSC2-derived secretome in the organic VC model, the Tohoku hospital pediatrics-1 (THP-1) monocyte adhesion to VSMCs at the specified experimental time points was assessed (Figure 4).

The time-dependent pattern of monocyte adhesion showed that under non-calcifying conditions (C−), the adherence of THP-1 cells remained relatively stable throughout the experimental period, ranging from approximately 15 to 60 cells per well across all time points. Compared with the (C−) group, significantly more THP-1 cells adhered to VSMCs cultured in the calcifying medium (C+) (*p* < 0.05), particularly at days 14 and 21, when the mean number of adherent cells reached 64 and 94 cells, respectively. In the group cultured with a calcifying medium enriched with the HATMSC2 secretome (C+S50+), THP-1 adhesion was significantly attenuated. On day 14, the mean number of adherent cells in the C+S50+ group was 39 compared to 64 cells in the C+ group. On day 21, the mean number of adherent cells in the C+S50+ group was significantly lower than in the C+ group and revealed 64 vs. 94 cells, respectively (*p* < 0.01).

### 3.4. HATMSC2-Derived Secretome Reduces mRNA Expression of Osteogenic Markers in VSMCs

**Figure 5:**
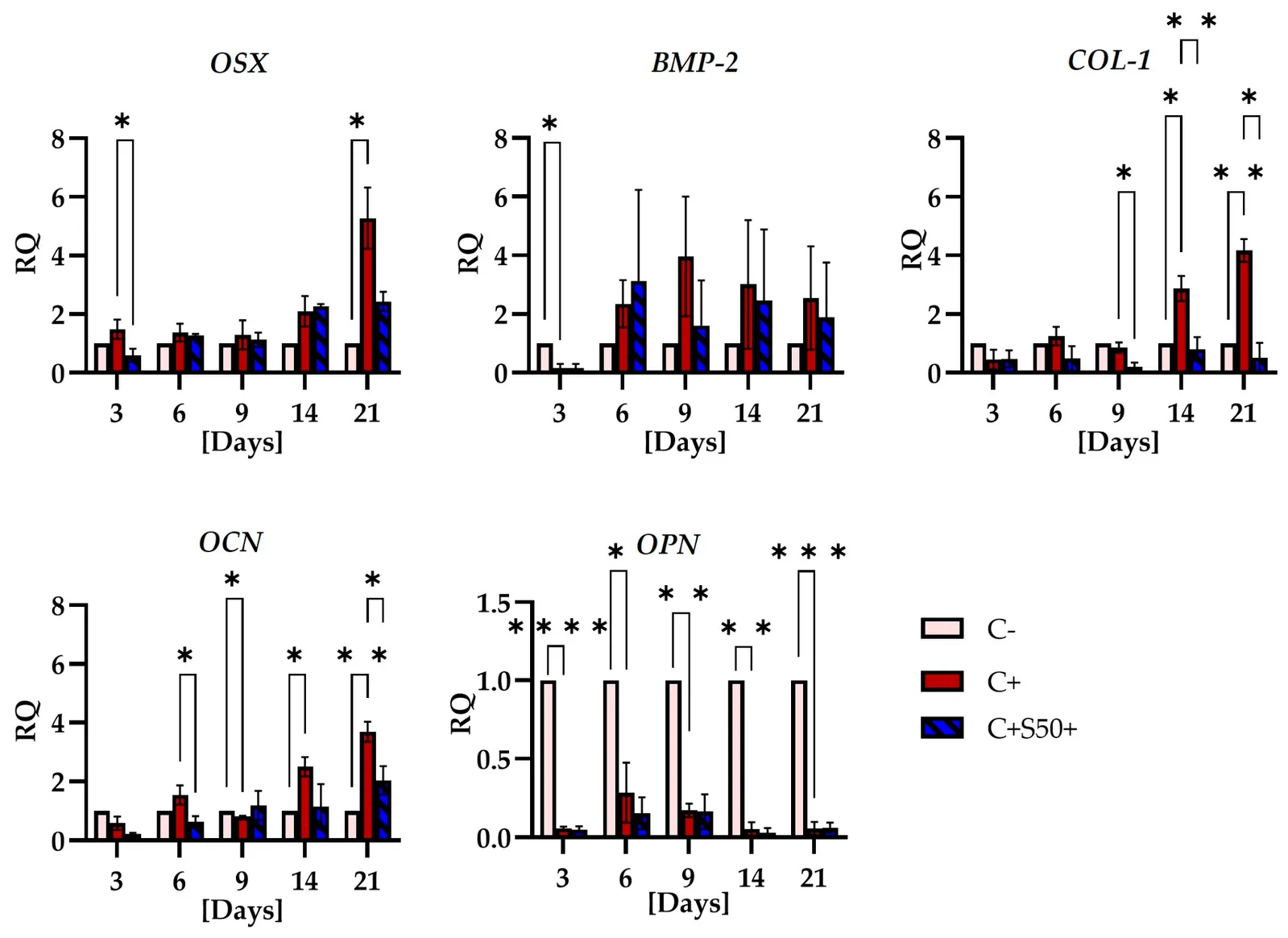
**Relative mRNA expression of osteogenic markers (*OSX*, *BMP-2*, *COL-1*, *OCN*, and *OPN*) in VSMCs assessed with real-time PCR.** Cells in a basal culture medium (C−), cells in an organic calcification medium (C+), and cells in a calcification medium enriched with the HATMSC2 secretome (C+S50+). Samples were analyzed in three independent experiments (*n* = 3), each in triplicate. Data represent mean ± SD, * *p* < 0.05, ** *p* < 0.01, *** *p* < 0.001, **** *p* < 0.0001. Values were considered significantly different at *p* < 0.05. Statistical significance was compared to C+.

Analysis of osteogenic marker expression revealed increased mRNA levels of all investigated markers except *OPN* in VSMCs cultured in the calcifying medium (C+), particularly on day 21 of the experiment (Figure 5). A time-dependent, moderate increase in the expression of *OSX*, *BMP-2*, *COL-1*, and *OCN* was observed in the C+ group compared to the C− group, which consisted of VSMCs cultured in the basal culture medium. Notably, *OPN* expression remained consistently significantly lower under calcifying conditions at all analyzed time points. Interestingly, the addition of the HATMSC2 secretome to the calcification medium reduced the relative quantification (RQ) of mRNA levels of all investigated markers. On day 3, *OSX* expression was significantly reduced in the secretome-treated group (C+S50+) (RQ = 0.59) compared to C+ (RQ = 1.49, *p* < 0.05). Analyses of later time points also showed a downregulation trend of *OSX* following secretome treatment. In particular, on day 21, *OSX* expression was lower in the C+S50+ group than in the C+ group (RQ = 2.44 vs. 5.28, respectively); however, it was not statistically significant. In contrast, *COL-1* expression was significantly downregulated in the secretome-treated group (C+S50+) compared with the C+ group from day 9 (RQ = 0.86 vs. 0.20, *p* < 0.05) until the end of the experiment (RQ = 4.17 vs. 0.51, *p* < 0.05). The HATMSC2 secretome suppressed *OCN* expression under calcifying conditions, with the strongest and statistically significant reductions observed on days 6 (RQ = 1.55 vs. 0.63) and 21 (RQ = 3.69 vs. 2.04).

### 3.5. Effects of HATMSC2 Secretome on RUNX2 and OPN Protein Expression and Cytoskeletal Organization in VSMCs Under Calcifying Conditions

**Figure 6:**
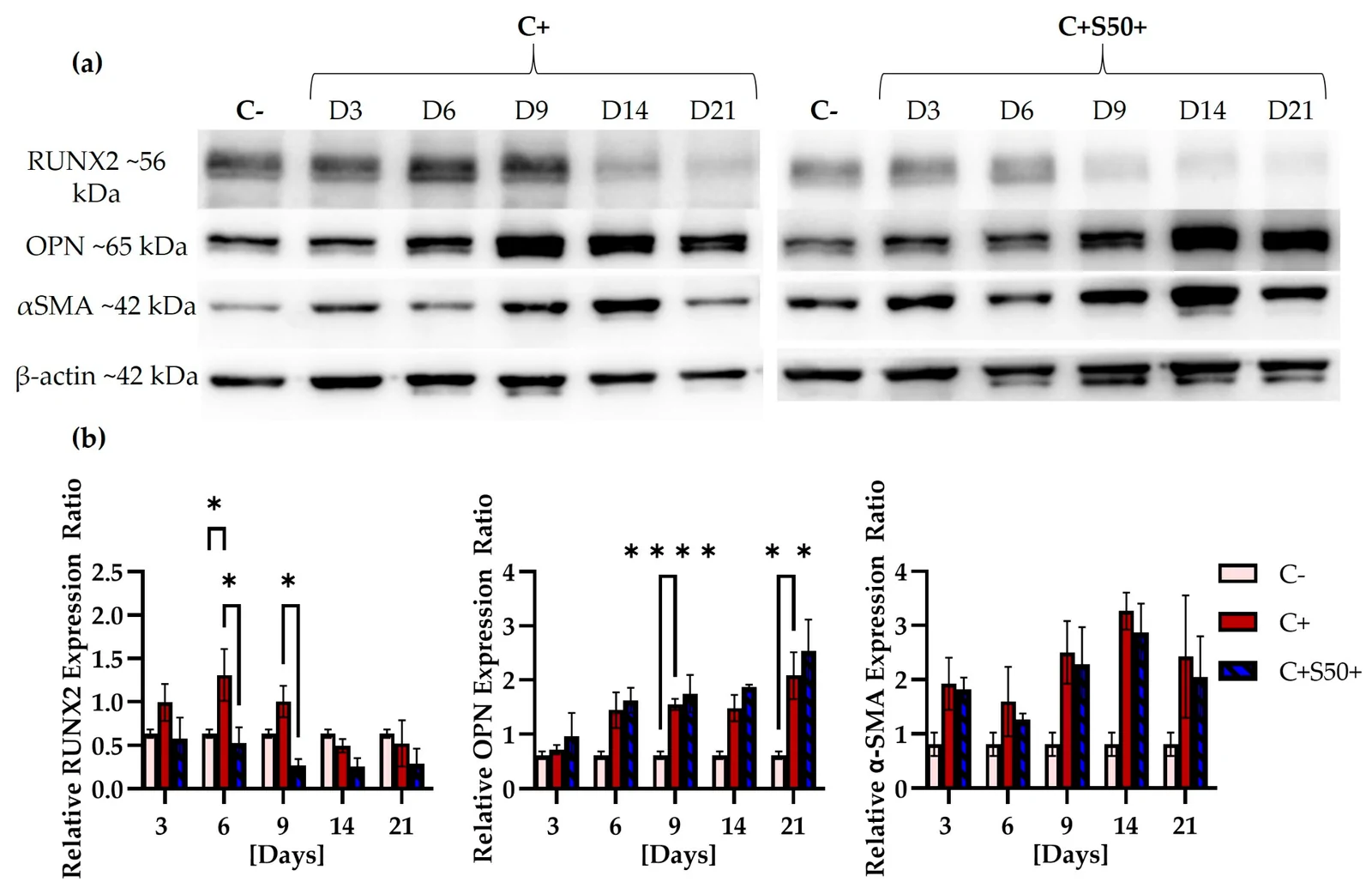
**Time-course analysis of RUNX2, OPN, and α-SMA expression in a vascular calcification model in vitro.** (**a**) Representative Western blot showing protein expression levels of RUNX2, OPN, and α-SMA over a 21-day period (days 3, 6, 9, 14, and 21). VSMCs were cultured in a basal medium (C−), organic calcifying medium alone (C+) or calcifying medium supplemented with the HATMSC2 secretome (C+S50+). (**b**) A densitometric analysis of Western blot results representing the relative expression ratios of RUNX2, OPN, and α-SMA. β-actin serves as the loading control and was used for densitometric normalization. Data represent mean ± SD, *n* = 3 (OPN, RUNX2) or *n* = 2 (α-SMA), * *p* < 0.05, ** *p* < 0.01, **** *p* < 0.0001. Values were considered significantly different at *p* < 0.05. Statistical significance was compared to C+.

The relative levels of RUNX2 and OPN were assessed using Western blot analysis (Figure 6a,b) after 3, 6, 9, 14, and 21 days of VSMCs culture under the following conditions: basal culture medium (C−), organic calcifying medium (C+), and organic calcifying medium supplemented with 50 µg/mL of the HATMSC2-derived secretome (C+S50+).

To assess the initiation of VSMCs osteogenic transdifferentiation, RUNX2 expression was monitored over a 21-day period. Under non-calcifying conditions (C−), the relative level of RUNX2 remained stable, at approximately 0.63-fold. In contrast, in the organic calcifying medium (C+), an early upregulation of RUNX2 was observed, peaking at day 6 (approximately 1.31-fold). The calcifying medium supplemented with the HATMSC2 secretome (C+S50+) abrogated this increase at day 6, maintaining RUNX2 levels close to the baseline. At day 9, RUNX2 levels declined across all groups; however, the values in the C+S50+ group were significantly lower than in the C+ group (approx. 0.27 vs. 1.00, respectively; *p* < 0.05) (Figure 6b). By days 14 and 21, RUNX2 expression remained low and relatively stable, across all analyzed groups.

Following the induction of calcification, a gradual increase in OPN expression was observed over the calcification period in the C+ group, starting from 0.72 at day 3 and reaching 2.08 at day 21. Compared with the C+ group, treatment with HATMSC2 secretome did not reduce OPN expression at any of the analyzed time points. The only significant differences were observed between the non-calcifying (C−) and calcifying (C+) groups at days 9 and 21 of the experiment.

Analysis of the α-SMA expression profile showed a decrease in calcifying control (C+) at days 3 and 6, reaching the values 1.92 and 1.59, respectively, compared with the C− group (0.81) (Figure 6b). In the cultures exposed to the calcifying medium supplemented with the HATMSC2 secretome (C+S50+), α-SMA levels on day 3 and 6 were slightly lower (reaching a value of 1.82 and 1.26, respectively) than in the C+ group. The relative amount of α-SMA was at a similar level on day 9 for all analyzed groups. On day 14, an increase in α-SMA levels was observed in the C+ and C+S50+ groups compared to the C− group (3.27, 2.87, and 0.81, respectively). However, on day 21, α-SMA expression decreased in the C+ and C+S50+ groups.

**Figure 7:**
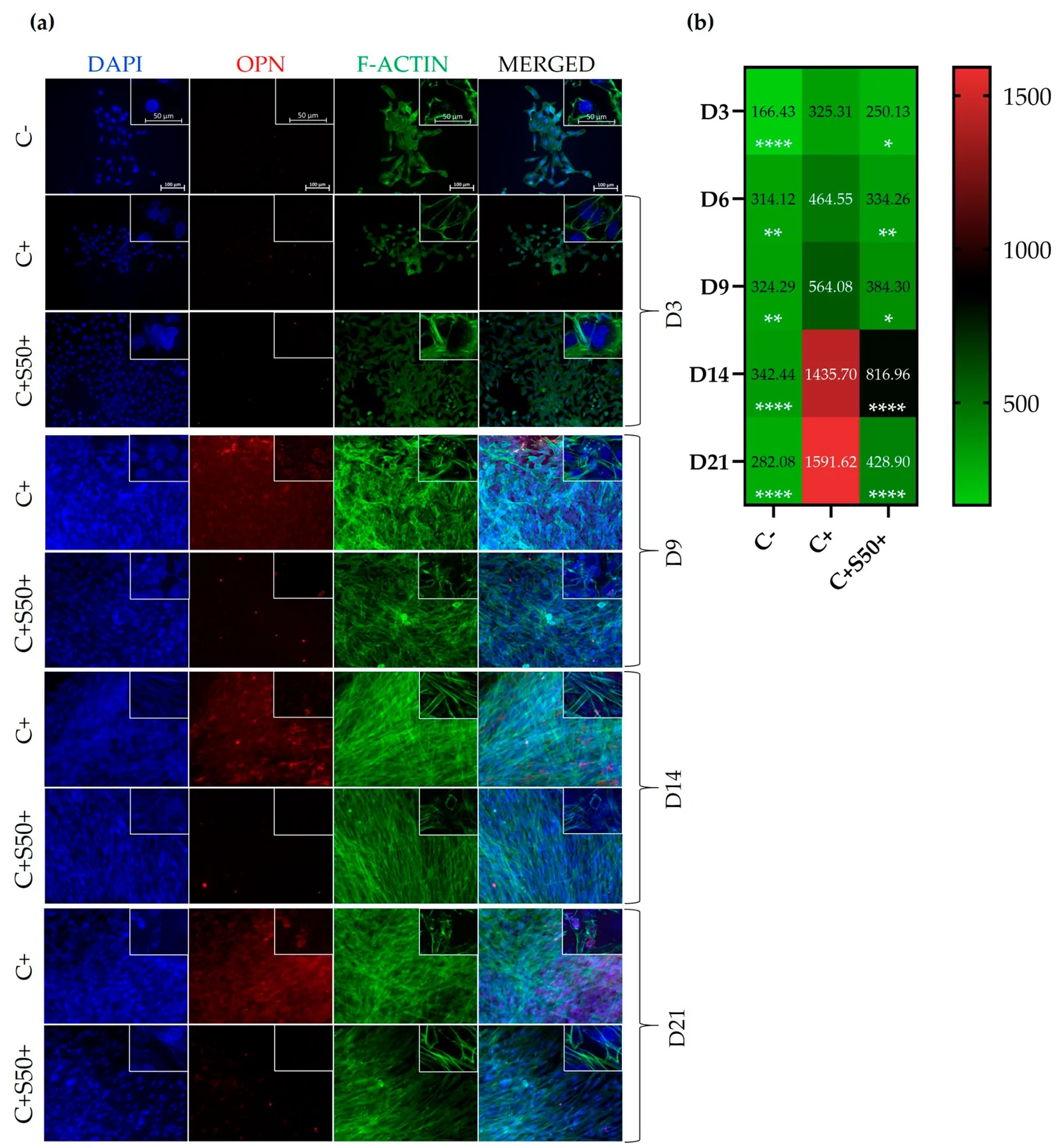
**Osteopontin expression in a vascular calcification model.** (**a**) Immunofluorescent staining of VSMCs cultured in: basal culture medium (C−), organic calcification medium alone (C+), and supplemented with an HATMSC2-derived secretome at a final concentration of 50 µg/mL (C+S50+). (**b**) Heatmap of mean fluorescent intensity (MFI) of OPN staining. The staining was performed in three independent experiments (*n* = 3), each in duplicate. Data represent mean ± SD, *n* = 3, * *p* < 0.05, ** *p* < 0.01, **** *p* < 0.0001. Values were considered significantly different at *p* < 0.05. Statistical significance was compared to C+. Immunofluorescence staining with magnification 10× and insert (magnification 63×). The scale bars presented in the upper images are used for all representative photographs.

Immunofluorescence staining revealed an increase in OPN expression over time in the C+ group compared to the control group (C−) (Figure 7a). Interestingly, reduced OPN expression was also observed in the group where the VSMCs were cultured in an organic calcifying medium enriched with the HATMSC2-derived secretome at a concentration of 50 µg/mL. Mean fluorescent intensity (MFI) confirmed an increase in OPN expression, especially at later time points, where MFI for C+ was assessed at 1435.70 (SD = 53.77) after 14 days and at 1591.62 (SD = 116.81) after 21 days of cell culture (Figure 7b). Interestingly, MFI for the C− group remained within the range of 166–342 throughout the experiment (SD = 30.20–53.29). Moreover, the addition of the HATMSC2 secretome to the organic calcification medium clearly reduced the expression of OPN throughout the experiment since day 3, where the MFI of the C+ group was 325.31 (SD = 41.72) compared to MFI = 250.13 in the C+S50+ group (SD = 35.13, *p* < 0.05). The greatest difference was seen after 21 days, where MFI for C+ was 1591.62 (SD = 116.81), and C+S50+ was assessed at 428.90 (SD = 157.99, *p* < 0.0001).

### 3.6. STRING- and Literature-Guided Screening and Quantitative Validation of Potential Anti-Calcification-Related Proteins in HATMSC2-Derived Secretome

**Figure 8:**
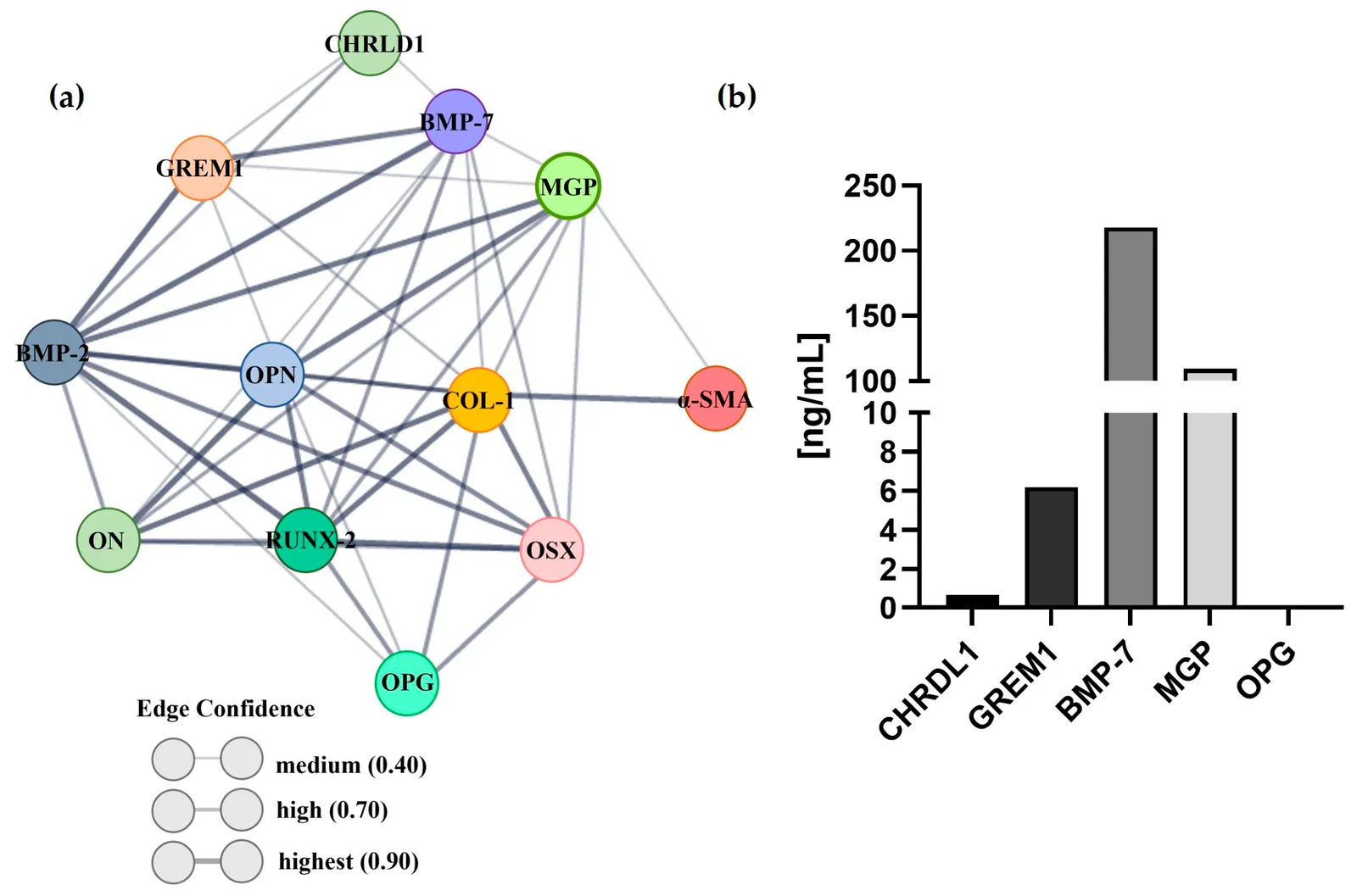
**STRING-based identification and quantification of anti-calcification proteins in HATMSC2 secretome.** (**a**) Protein–protein interaction network of the analyzed factors associated with vascular calcification. The network visualization was generated using the STRING software, v12.0. Edges represent STRING functional associations between proteins contributing to a shared biological function and do not necessarily indicate direct physical interactions. In confidence mode, edge thickness reflects the confidence of each association based on the available evidence, with thicker lines indicating higher confidence rather than greater biological interaction strength. (**b**) Quantification of CHRDL1, GREM1, BMP-7, MGP, and OPG in the HATMSC2 secretome with ELISA. Abbreviations: α-SMA—alpha-smooth muscle actin; COL-1—collagen 1; MGP—matrix Gla protein; OSX—osterix; ON—osteonectin; OPN—osteopontin; OPG—osteoprotegerin.

An with the silico, a STRING-based protein network analysis (Figure 8a), together with a targeted literature review, was used to identify proteins potentially involved in the regulation of vascular calcification. STRING analysis identified gremlin 1 (GREM1), matrix gla protein (MGP), and chordin-like protein 1 (CHRDL1) as proteins potentially involved in vascular calcification. In parallel, a targeted literature review revealed additional factors involved in the regulation of vascular calcification, including BMP-7 [39,40,41] and osteoprotegerin (OPG) [42,43], which prompted their inclusion in the analysis panel.

These five candidate proteins were subsequently assessed in the HATMSC2-derived secretome using ELISA. Four proteins were detectable and quantified, namely, CHRDL1, GREM1, BMP-7, and MGP. The concentration of MGP was 109.50 ± 3.88 ng/mL, BMP-7 was 217.92 ± 14.39 ng/mL, GREM1 was 6.21 ± 0.46 ng/mL, and CHRDL1 was 0.70 ± 0.004 ng/mL. In contrast, OPG levels were below the assay detection limit (Figure 8b).

## 4. Discussion

The present study demonstrates that the HATMSC2-derived secretome attenuates osteogenic differentiation of VSMCs under calcifying conditions. This effect was associated with reduced calcium deposition, downregulation of osteogenic markers, attenuated THP-1 adhesion, and the presence of several anti-calcification-related proteins within the HATMSC2 secretome.

### 4.1. Comparative Analysis and Calcium Accumulation Across VC Models

We began our investigation by evaluating the effects of the HATMSC2 secretome in two distinct in vitro models of VC, focusing on the extent of calcium deposit formation. In both models, based on inorganic and organic phosphate donors, treatment with the secretome reduced calcium deposition. As presented in this study, for the inorganic phosphate model (2 mM Pi), the HATMSC2 secretome induced only modest, non-significant reductions in calcium deposition despite clear trends toward decreased mineralization at days 11 and 14. This contradicts the findings of Guo et al. [23] and Lou et al. [24], who reported statistically significant reductions in both calcium content and ALP activity in models characterized by high inorganic phosphate concentrations (2.5 mmol/L Pi) following treatment with BMSC-derived exosomes (100 μg/mL) over seven days. The differential efficacy between our inorganic model and the results reported by other authors is likely related to differences in the phosphate concentration used, as well as other anti-calcifying factors and their concentrations (exosomes from BMSC vs. full secretome from HATMSC2).

Interestingly, this reduction was statistically significant relative to the C+ group only in the organic model. In the context of the organic phosphate calcification model, this finding contrasts with the classic inorganic phosphate calcification model, where pronounced mineral deposition often coincides with a substantial loss of cell viability, limiting the window for interrogating dynamic molecular events. These discrepancies likely reflect fundamental differences in calcification mechanisms: inorganic phosphate-driven calcification is dominated by relatively passive mineral precipitation coupled with rapid cell death, whereas organic models engage more active, cell-mediated transdifferentiation processes that are inherently more susceptible to paracrine modulation by secretome-derived factors. The use of a medium enriched with β-GP, ascorbic acid, and dexamethasone has a milder and more biologically relevant effect on VSMCs than the direct administration of inorganic phosphate (Pi). β-GP requires an enzymatic breakdown by ALP into free phosphate, which induces active phenotypic transdifferentiation of the cells. In contrast, the administration of Pi supplies phosphate directly, triggering rapid mineralization and apoptosis. Ascorbic acid facilitates COL-1 synthesis, while dexamethasone activates transcription factors (e.g., RUNX2) that control the osteochondrogenic reprogramming of VSMCs. An osteogenic medium activates protective mechanisms that promote cell growth and inhibit pathological mineralization. In the Pi model, the cells die before they can initiate these adaptive processes [44]. In light of this, we focused on an organic calcification medium based on beta-glycerophosphate, supplemented additionally with ascorbic acid and dexamethasone. This model offers a more suitable platform for analyzing time-dependent biological changes in the calcification process, including transcriptional reprogramming and matrix remodeling, thereby enabling a more reliable assessment of the secretome’s protective and anti-calcification properties of HATMSC2. Moreover, our findings demonstrate that the HATMSC2 secretome significantly attenuates both calcium deposition ALP activity in an organic calcification model, aligning with observations from BMSC-derived secretome studies [25] while revealing important model-dependent differences in therapeutic efficacy. In the organic calcification model, HATMSC2 secretome treatment (C+S50+, 50 μg/mL) resulted in a sustained reduction in calcium deposits throughout the 21-day experimental period. This protective effect was paralleled by marked suppression of ALP activity, with C+S50+-treated cells exhibiting enzyme levels comparable to non-calcifying controls at day 21.

These results are consistent with Wang et al. [26], who demonstrated that a BMSC-derived conditioned medium effectively reduced both calcium content and ALP activity in VSMCs cultured with 10 mM β-glycerophosphate over seven days. Similarly, Wang et al. [25] reported significant reductions in ALP activity and osteogenic marker expression (*BMP-2*, *RUNX2*) in VSMCs treated with the BMSC secretome in a 14-day β-GP model. Our organic model revealed gradual calcium accumulation and sustained secretome efficacy compared to simpler β-GP-only models, suggesting that the multi-component organic stimulus better recapitulates the complex microenvironment of pathological VC.

Taken together, our data demonstrate that the HATMSC2 secretome exerts potent anti-calcification effects comparable to those reported for the BMSC secretome, with ALP activity suppression and calcium accumulation inhibition being the most pronounced and statistically robust in the organic calcification model. The sustained efficacy observed through day 21, combined with preserved VSMC viability that enabled the comprehensive analysis of osteogenic markers, positions the organic calcification model as a superior platform for evaluating cell-free therapeutic strategies targeting VC.

### 4.2. Early Osteogenic Markers: OSX, BMP-2, RUNX2, and COL-1

Our study demonstrates that supplementation of the calcifying medium with the HATMSC2 secretome suppressed key early osteogenic regulators, i.e., *OSX*, *COL*-*1*, and *RUNX2*, and reduced mineralization, thereby disrupting the transition toward an osteoblast-like phenotype that underlies pathological VC. This is in line with a previous work on a BMSC-derived conditioned medium, which reduced calcium deposition and osteogenic marker expression (*RUNX2*, *OCN*) in VSMCs [25]. A key observation from our study is that the HATMSC2 secretome effectively reduced *OSX* mRNA levels; the level was significantly lower as early as day 3, although—despite a clear downward trend observed up to day 21—the differences did not reach statistical significance. This pattern suggests an early and persistent modulatory effect on *OSX*, a *RUNX2*-dependent transcription factor central to osteoblast differentiation, thereby limiting the phenotypic switching of VSMCs to an osteoblast-like profile [45,46,47,48].

Consequently, the relative content of the RUNX2 protein reached a distinct peak on day 6, initiating the osteogenic cascade and preceding extensive matrix mineralization [49,50]. In contrast, the addition of HATMSC2 secretome markedly reduced the day-6 RUNX2 surge and maintained lower RUNX2 levels at day 9, at which point ALP activity was already reduced to values comparable with the non-calcifying control. Together, these findings indicate that the secretome intercepts the early osteogenic program at both the protein level (RUNX2) and functional (ALP) levels, limiting the emergence of an osteoprogenitor-like phenotype before irreversible mineral deposition occurs. The level of *COL-1* mRNA was significantly lower in the secretome-treated group than in the C+ group from day 9 onwards and remained suppressed up to day 21, indicating sustained attenuation of pathological extracellular matrix remodeling. This prolonged reduction is particularly favorable in the context of vascular health, as excessive *COL-1* expression contributes to increased vessel wall stiffness and calcification [51]. Additionally, it has been found that *COL-1* serves not only as a scaffolding structure for mineralization, but as a signaling matrix as well, whereby it guides the distribution, clustering, and crystallization of calcification vesicles and induces the change in the phenotype of VSMCs into osteochondral cells [51]. Therefore, suppression of *COL-1* expression by the HATMSC2-derived secretome may counteract key molecular events that drive the progression of pathological VC and its associated cardiovascular complications.

*BMP*-*2* induces osteogenesis-related matrix proteins, such as OPN and COL-1, which reorganize the extracellular matrix and facilitate calcium-related processes [52]. An apparent discrepancy in our data is that *BMP*-*2* mRNA did not show significant overall suppression despite pronounced reductions in RUNX2 protein and ALP activity. However, BMP-2-dependent signaling is regulated at multiple levels, including ligand availability, receptor expression, Smad activation, and post-transcriptional mechanisms, and is not fully captured by transcript abundance alone [52]. In our experimental model, calcification-promoting conditions induced a moderate, time-dependent increase in BMP-2 mRNA, whereas the effect of the secretome contributed primarily to the downregulation of RUNX2 and ALP expression. However, the impact on downstream nodes of the signaling pathway remains to be investigated. Because RUNX2 integrates inputs from several osteogenic pathways (BMP/Smad, Wnt/β-catenin) [53], its suppression may occur without a parallel decrease in BMP-2 transcript. In light of the above, the lack of a marked BMP-2 mRNA change does not contradict the anti-calcification effect. The present study did not directly assess BMP receptor activation, Smad1/5/8 phosphorylation, or other downstream signaling events, and the results presented by us do not establish that HATMSC2-derived secretome acts through the BMP/RUNX2 axis. However, our data indicate that secretome treatment is associated with reduced expression of selected osteogenic markers, including OPN and RUNX2, under calcifying conditions. The reduction in RUNX2 may reflect the modulation of one or more osteogenic regulatory pathways, including, but not limited to, BMP-related signaling. A direct assessment of pathway activity will be required to define the molecular basis of this effect.

### 4.3. α-SMA Expression and Preservation of the VSMC Phenotype

Data regarding α-SMA levels in the C+ group showed only a modest early decrease compared with non-calcifying conditions. Previously, Deladillo et al. [54] reported that oxidative stress and increased expression of the enzyme glutamate-cysteine ligase (GCL) drove the loss of the contractile phenotype (decreased expression of α-SMA) and impaired VSMC function. Our data do not clearly indicate a sustained decrease in α-SMA expression; however, they align with the observations by Patel et al. [49], who reported that classic VSMC markers, such as α-SMA, may be partially retained in calcifying VSMCs or even maintained in osteoblast-like cells. This suggests an intermediate, hybrid phenotype, rather than a complete transition to a fully osteoblastic phenotype. At the same time, subtle, time-related fluctuations in α-SMA observed in our study likely reflect cytoskeletal dysregulation and a stress-related remodeling process that precedes overt functional failure under calcifying conditions [55].

An early activation of TGF-β signaling and pronounced oxidative stress have been shown to strongly and rapidly stimulate the ACTA2 promoter, which encodes α-SMA [56], potentially accounting for the transient changes in α-SMA levels observed on days 6 and 9/14 in the groups analyzed in this study. It seems possible that the HATMSC2 secretome helps to maintain VSMCs in a state resembling their physiological contractile phenotype. This protective effect is likely related to its complex composition, rich in antioxidant and anti-inflammatory paracrine factors, which may counteract the oxidative stress–GCL axis described previously [54], thereby limiting both contractile impairment and osteogenic transdifferentiation, especially at an early stage of calcification process.

### 4.4. Late Osteogenic Markers: OCN and OPN

The analysis of late osteogenic markers, namely OCN and OPN, supports the findings, that HATMSC2 secretome may effectively intervene in the pathological transdifferentiation process of VSMCs. In the calcifying medium, we observed a significant, time-dependent increase in mRNA relative expression of *OCN*, which reflects progression toward a more advanced stage of osteogenic differentiation and matrix mineralization during the calcification process. This pattern is in line with the notion that calcifying VSMCs adopt an incomplete osteoblast-like phenotype [49] characterized by increased expression of osteogenic genes and early mineral deposition.

In contrast to *OCN*, we did not observe any significant change in *OPN* mRNA expression in the presence of the HATMSC2 secretome compared with the C+ group over the experimental period. Interestingly, in the C+ group, we observed lower OPN mRNA expression, whereas Western blot analysis showed a higher relative abundance of OPN in C+ compared with the C− group. This discrepancy between the reduced *OPN* mRNA level and the increased amount of OPN protein in the C+ group compared to the C− group may be explained, at least in part, by post-transcriptional, transcriptional, and post-translational regulatory mechanisms, as well as by differences in the temporal dynamics of OPN transcription, translation, secretion, and turnover [57,58,59].

### 4.5. Immunomodulatory Activity of HATMSC2 Secretome

In addition to suppressing osteogenic differentiation, HATMSC2-derived secretome markedly reduced THP-1 monocyte adhesion to calcifying VSMCs, suggesting attenuation of the pro-inflammatory microenvironment associated with VC.

Numerous studies have shown that monocyte adhesion to vascular cells is a critical early event in the development of vascular inflammation and calcification, facilitating cell differentiation into macrophages, immune cell infiltration, release of proinflammatory cytokines, and the propagation of local inflammatory responses [60,61,62,63]. Our observation is in line with previous studies demonstrating that MSC-derived secretomes exert anti-inflammatory effects by reducing inflammatory signaling and limiting the expression of mediators that promote vascular calcification [26].

### 4.6. Potential Proteins Associated with the Attenuation of Vascular Calcification Identified in the HATMSC2 Secretome

The anti-calcification potential of MSC-derived secretomes has been demonstrated both in the present study and in previous reports. However, the specific bioactive components responsible for these protective effects remain largely undefined. To date, most mechanistic studies have focused on extracellular vesicle-associated microRNAs as the principal mediators of MSC paracrine activity. For example, recent studies have identified specific exosomal microRNAs, including miR-381-3p and miR-335, as the key mediators of the anti-calcification effects of BMSC-derived exosomes through the regulation of the NFAT5 and SP1/RUNX2 signaling pathways, respectively [35,36]. In contrast, considerably less attention has been paid to soluble protein factors that may also contribute to the anti-calcification properties of the MSC secretome. Since our immortalized HATMSC2 cell line has previously been shown to secrete a broad spectrum of biologically active proteins [30,33], we sought to identify protein components potentially responsible for the anti-calcification effects observed in the present study. Importantly, Wang et al. [25] demonstrated that BM-derived MSC-CM attenuates VSMC calcification by inhibiting the BMP-2/Smad1/5/8 signaling pathway, highlighting BMP signaling as a central target of MSC-mediated anti-calcification activity. This finding provided the rationale for focusing our bioinformatic analysis on proteins capable of modulating BMP-related pathways. To achieve this, we combined STRING network analysis with a targeted literature review, which identified GREM1, MGP, CHRDL1, BMP-7, and OPG as potential candidates for subsequent experimental validation.

Quantitative ELISA confirmed the presence of GREM1, MGP, CHRDL1, and BMP-7 in the HATMSC2 secretome. However, their individual or combined functional contribution was not examined in the present study. Importantly, the concentrations measured by ELISA correspond to the concentrated secretome and not to the final concentrations of individual factors in the culture medium. Therefore, these concentrations cannot be directly compared with those used in studies investigating individual factors. GREM1 and CHRDL1 are extracellular BMP antagonists that negatively regulate BMP-dependent signaling within the TGF-β superfamily [64,65], whereas MGP is a well-characterized inhibitor of vascular mineral deposition [15]. The coexistence of proteins related to the attenuation of vascular calcification with the observed early suppression of RUNX2 and ALP highlights BMP signaling as a pathway potentially associated with the anti-calcification effects of the HATMSC2 secretome and warrants further investigation. Because BMP-2 is an upstream driver of osteogenic transdifferentiation, secretome-derived antagonists that blunt BMP-2 signaling have the potential to simultaneously dampen multiple downstream osteogenic outputs, including RUNX2 activation, ALP induction, and pathological matrix remodeling, thereby interrupting the calcification cascade at an early, modifiable stage. Moreover, in HATMSC2-derived secretome, we detect BMP-7, which has been proposed to exert down-regulators of vascular calcification [39,40]. Importantly, because BMP-7 acts as an agonist of BMP signaling, rather than an inhibitor of the BMP axis, its reported anti-calcific effects should not be interpreted as evidence of BMP pathway inhibition. The final molecule we considered as a potential anti-calcification factor was OPG, a circulating glycoprotein that acts as a key negative regulator of cardiovascular calcification, protecting blood vessels and heart valves from abnormal hardening and mineral deposition [43,66]. Although Vašíček et al. [67] detected OPG in conditioned media from HATMSCs collected after 24 h, in our study, OPG concentrations in the HATMSC2 secretome were below the ELISA detection limit. Importantly, this result should not be interpreted as definitive evidence that HATMSC2 does not produce OPG. Rather, it may indicate that OPG secretion was low under the culture conditions used in the present study or that its concentration was below the analytical sensitivity of the assay. While these divergent findings may be attributable, at least in part, to methodological differences, they are most likely attributable to methodological differences, as Vašíček et al. used a semi-quantitative fluorescence-based assessment of protein spots, whereas we applied a quantitative ELISA approach, which may impose stricter sensitivity thresholds and highlight the lower abundance of OPG in our system. In addition, OPG production may be influenced by the cellular source and state, culture conditions, duration of conditioning, and exposure to inflammatory or osteogenic stimuli. Future studies should, therefore, assess whether OPG release from HATMSC2 changes under calcification- or inflammation-mimicking conditions and should consider the use of more sensitive analytical methods.

### 4.7. Clinical Potential

The production of proteins using established cell lines is now a standard practice in biotechnology and the biopharmaceutical industry, mainly due to their high stability, reproducibility and the possibility of large-scale cultivation. Unlike primary cells, cell lines exhibit an unlimited or significantly extended capacity for proliferation, which enables high production yields to be achieved while maintaining uniform culture conditions. Furthermore, they are characterized by lower biological variability, which translates into greater process reproducibility and a more predictable quality of the final product [68]. An additional advantage of established cell lines is the ability to genetically modify them easily and to select clones with high levels of protein expression. This allows for the optimization of production parameters and the production of proteins with appropriate post-translational modifications, such as glycosylation, which is crucial for the biological activity of many therapeutic proteins [69]. Primary cells, although they better reflect the physiological properties of tissues, have a limited number of divisions, exhibit significant heterogeneity, and are more demanding in terms of culture conditions. These characteristics hinder the standardization of the production process and limit the potential for using primary cells in the large-scale production of recombinant proteins. Consequently, established cell lines remain the preferred expression system in both scientific research and the industrial production of biopharmaceuticals [70]. With this in mind, the use of the secretome from our HATMSC2 cell line is not only more suitable in terms of reproducibility and increased versatility, but also represents a promising candidate for future therapeutic applications. In this study, we have demonstrated for the first time that the secretome derived from HATMSC2 cells exerts a significant protective effect against VC. Our results, combined with previously published data, indicate that the HATMSC2 secretome exhibits a stronger regenerative effect in vitro than other conditioned media derived from primary MSCs. This confirms its potential translational relevance as a standardized, cell-free source of growth factors, cytokines, and soluble proteins. The data obtained on the anti-calcification properties of the HATMSC2 secretome provide new insights into its clinical potential as the basis for a potentially applicable cell-free therapeutic approach capable of both modulating pathological vascular calcification and supporting tissue repair processes. These properties make the HATMSC2 secretome a promising candidate for the development of new therapeutic strategies aimed at counteracting vascular calcification, complementing its established role in regenerative medicine applications.

### 4.8. Strengths and Limitations

A major strength of this study is the comprehensive characterization of the anti-calcification properties of the HATMSC2-derived secretome. In addition to demonstrating, for the first time, that this immortalized MSC line exhibits anti-calcification activity, we combined functional, molecular, and bioinformatic approaches to identify protein candidates that may contribute to these protective effects.

However, although we have identified several potential molecules that are associated with the attenuation of VC, we did not evaluate BMP receptor activation, Smad1/5/8 phosphorylation, or RUNX2 nuclear localization. Moreover, while GREM1, BMP-7, CHRDL1, and MGP were detected in the HATMSC2-derived secretome, their causal contribution to the observed anti-calcification effects remains unknown. Future studies employing neutralizing antibodies or gene-silencing approaches will be required to determine the individual and combined roles of these proteins in mediating the anti-calcification activity of the HATMSC2-derived secretome.

Furthermore, the present study did not include a comprehensive profiling of the HATMSC2 secretome (e.g., proteomics, extracellular vesicle, miRNA, or lipid profiling), nor a direct functional comparison with the primary MSC secretome. Although the non-tumorigenic properties of the HATMSC2 cell line have been previously demonstrated [30], further studies are warranted to comprehensively assess its safety and therapeutic potential. Finally, in vivo studies in a clinically relevant animal model of vascular calcification will be required to further validate the anti-calcification effects of the HATMSC2 secretome.

## 5. Conclusions

In conclusion, the HATMSC2-derived secretome effectively attenuated VSMCs in an organic calcification model in vitro, as demonstrated by reduced mineral deposition and ALP activity, suppression of osteogenic markers at both the mRNA (*OSX*, *BMP-2*, *COL-1*, *OCN*) and protein level (RUNX2), and reduction in THP-1 monocyte adhesion to calcifying VSMCs. A bioinformatic analysis combined with literature-guided screening enabled the identification of several potential anti-calcification molecules, including BMP-7, CHRDL1, and MGP. These proteins were detected in the HATMSC2-derived secretome and represent candidate factors that may contribute to its anti-calcification activity, potentially acting in concert with other secretome components, including extracellular vesicles and regulatory RNAs. Overall, our data reveal a previously unrecognized anti-calcification property of the immortalized HATMSC2 cell line, which extends the scope of its known regenerative potential and identifies it as a promising candidate for cell-free therapies targeting vascular calcification. Further mechanistic and functional studies are required to determine the signaling pathways involved and to define the contribution of individual secretome components.

## Figures and Tables

**Table 1:** Summary of experimental groups and abbreviations.

Group Name	Group Description
C−	VSMCs cultured in a basal culture medium
C+	VSMCs cultured in a calcifying medium
C+S50+	VSMCs cultured in a calcifying medium supplemented with 50 µg/mL of HATMSC2-derived secretome (S)

**Table 2:** Real-time PCR primer sequences.

Gene	Primer Sequences (5′-3′)	Fragment Size (bp)	GenBank Accession No.
*HPRT*	F: GAAGAGCTATTGTAATGACC R: GCGACCTTGACCATCTTTG	177	AC004383.1
*OSX*	F: CAGCTCTCTCCATCTGCCTG R: AGTTGTTGAGTCCCGCAGAG	164	AC073611.29
*BMP-2*	F: GAGGTCCTGAGCGAGTTCGA R: ACCTGAGTGCCTGCGATACA	123	AF040249.1
*COL-1*	F: AGGTGCTGATGGCTCTCCT R: GGACCACTTTCACCCTTGT	105	AC015909.14
*OCN*	F: GCAGGTGCGAAGCCCAGCGGTGCAGAG R: GGGCTGGGAGGTCAGGGCAAGGGCAAG	133	M34013.1
*OPN*	F: GCCGAGGTGATAGTGTGGTT R: AACGGGGATGGCCTTGTATG	149	D14813.1

## Data Availability

The original contributions presented in this study are included in the article/Supplementary Materials. Further inquiries can be directed to the corresponding author.

## Supplementary Materials

The following supporting information can be downloaded at: https://www.mdpi.com/article/10.3390/cells15211925/s1, Figure S1: Representative Western Blot of RUNX2, OPN, and α-SMA across four distinct reference cell lines; Figure S2: Quantification of Alizarin Red S staining by CPC method of inorganic (a) and organic (b) calcification models.

## Abbreviations

The following abbreviations are used in this manuscript:

Abs	absorbance
ALP	alkaline phosphatase
BMP-2	bone morphogenetic protein 2
BMP-7	bone morphogenetic protein 7
BMSCs	bone marrow-derived MSCs
CHRDL1	chordin-like protein 1
CKD	chronic kidney disease
COL-1	collagen type I
CPC	cetylpyridinium chloride
CPP	calciprotein particles
DMEM	Dulbecco’s modified Eagle medium
ENPP1	ectonucleotide pyrophosphatase/phosphodiesterase 1
FBS	fetal bovine serum
GCL	glutamate-cysteine ligase
GREM1	gremlin 1
HATMSCs	human adipose tissue-derived MSCs
HGF	hepatocyte growth factor
HPRT	hypoxanthine-guanine phosphoribosyltransferase
IGF-1	insulin-like growth factor 1
MEM	minimum essential medium
MGP	matrix gla protein
MMP	matrix metalloproteinases
MSCs	mesenchymal stem/stromal cells
NFAT5	nuclear factor of activated T-cells
OCN	osteocalcin
ON	osteonectin
OPG	osteoprotegerin
OPN	osteopontin
OSX	osterix
PBS	phosphate-buffered saline
PFA	paraformaldehyde
RQ	relative quantification
RT	room temperature
RUNX2	runt-related transcription factor 2
S	secretome
SM22α	smooth muscle protein 22α
SM-MHC	smooth muscle myosin heavy chain
SPP1	secreted phosphoprotein 1
SRB	sulforhodamine B
TGF-β	transforming growth factor-beta
THP-1	Tohoku hospital pediatrics-1
VC	vascular calcification
VEGF	vascular endothelial growth factor
VSMCs	vascular smooth muscle cells
α-SMA	smooth muscle α-actin
β-GP	β-glycerophosphate
