# Supplementary material for: Anti-Calcification Effects of Immortalized Human Adipose Tissue-Derived Mesenchymal Stem/Stromal Cell (HATMSC2) Secretome in an In Vitro Model of Vascular Calcification
Source: Cells. 2026 Oct 23;15(21):1925. doi: 10.3390/cells15211925 (PMC13762033; doi:10.3390/cells15211925)
Supplement: Supplementary file 1 [file cells-15-01925-s001.zip › cells-4595827-supplementary.pdf]

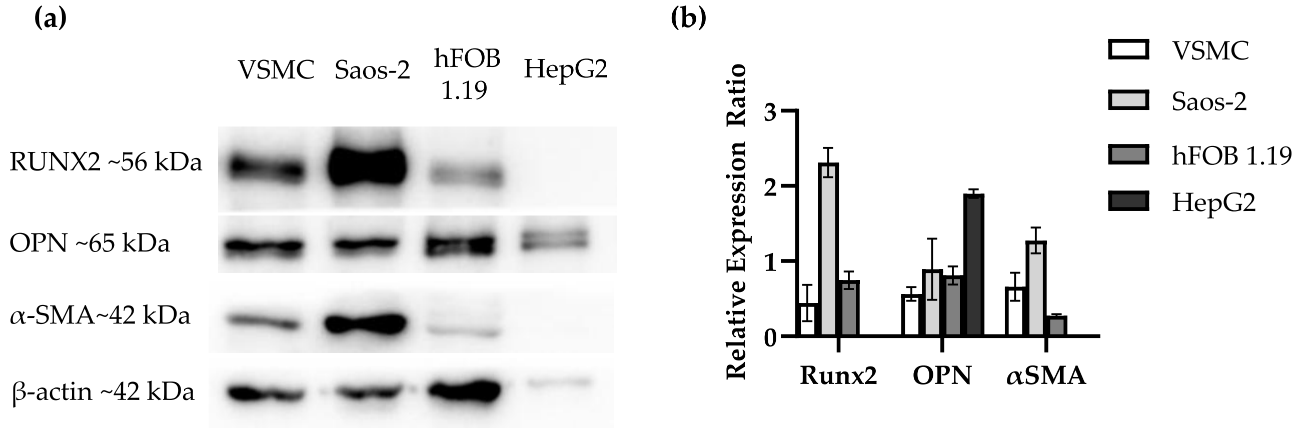

Figure S1. Representative Western Blot of RUNX2, OPN, and α-SMA across four distinct reference cell lines. Western blot (a) and relative protein expression ratios (b) of RUNX2, OPN, and α-SMA for: VSMCs, human osteosarcoma (Saos-2), human fetal osteoblasts (hFOB 1.19), and human hepatocellular carcinoma (HepG2). β-actin serves as the loading control. The analyzed samples were from two independent experiments (n = 2). Data are presented mean ± SD.

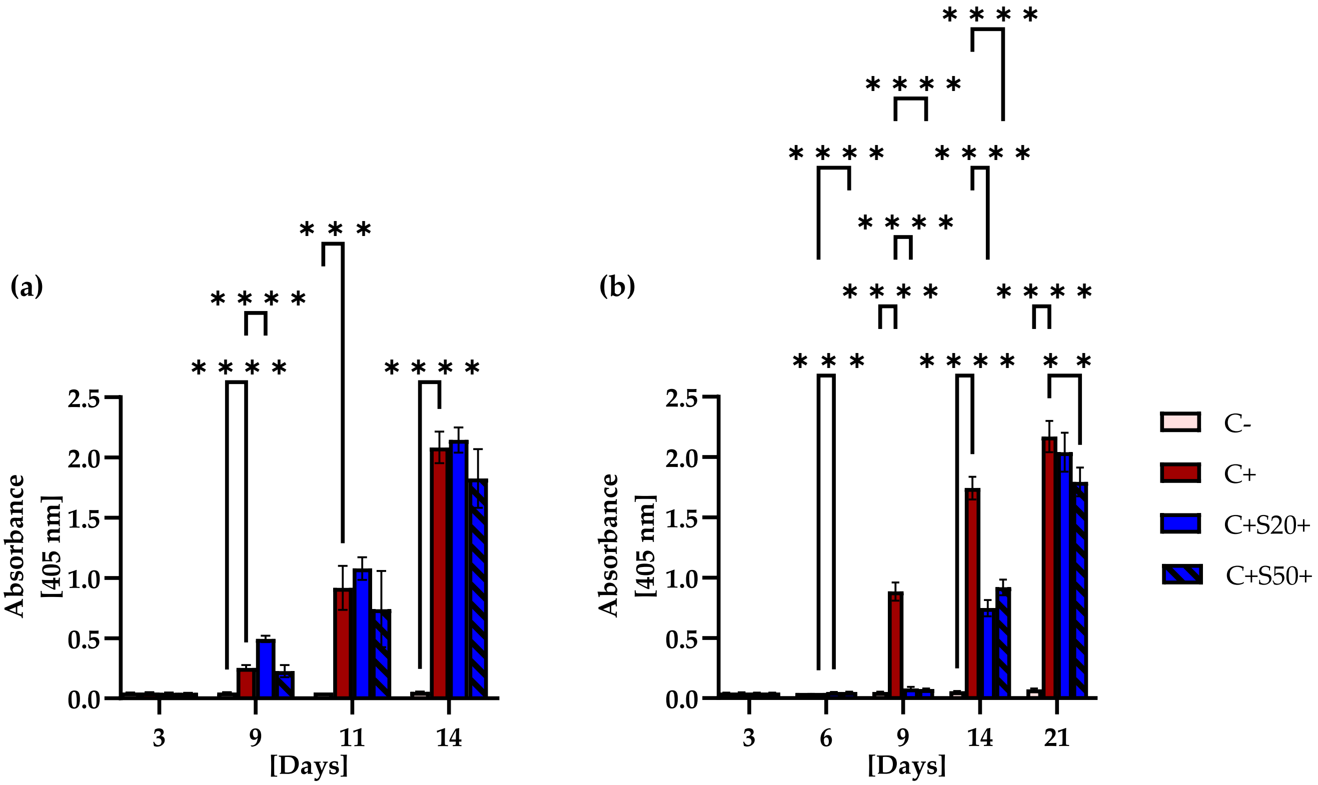

Figure S2. Quantification of Alizarin Red S staining by CPC method of inorganic (a) and organic (b) calcification models. Cells cultured in basal culture medium for each model (C-), cells cultured in organic calcification medium alone (C+) and enriched with HATMSC2 secretome in final protein concentration 20 µg/mL (C+S20+) and 50 µg/mL (C+S50+). The analyzed samples were from three independent experiments (n = 3), each in duplicate. Data are presented as mean ± SD, * $p < 0.05$, ** $p < 0.01$, *** $p < 0.001$, **** $p < 0.0001$. Statistical significance was compared to C+.
